# A JAR of Chirps: The Gymnotiform Chirp Can Function as Both a Communication Signal and a Jamming Avoidance Response

**DOI:** 10.3389/fnint.2019.00055

**Published:** 2019-10-02

**Authors:** Caitlin E. Field, Thiago Alexandre Petersen, José A. Alves-Gomes, Christopher B. Braun

**Affiliations:** ^1^Department of Psychology, Hunter College, The City University of New York, New York, NY, United States; ^2^New York City Department of Parks and Recreation, New York, NY, United States; ^3^Laboratório de Fisiologia Comportamental e Evolução, Instituto Nacional de Pesquisas da Amazônia, Manaus, Brazil

**Keywords:** electric fish, jamming avoidance response, hindbrain circuit, electric organ discharge, communication signals, pacemaker nucleus

## Abstract

The weakly electric gymnotiform fish produce a rhythmic electric organ discharge (EOD) used for communication and active electrolocation. The EOD frequency is entrained to a medullary pacemaker nucleus. During communication and exploration, this rate can be modulated by a pre-pacemaker network, resulting in specific patterns of rate modulation, including stereotyped communication signals and dynamic interactions with conspecifics known as a Jamming Avoidance Response (JAR). One well-known stereotyped signal is the chirp, a brief upward frequency sweep usually lasting less than 500 ms. The abrupt change in frequency has dramatic effects on phase precession between two signalers. We report here on chirping in *Brachyhypopmus* cf. *sullivani*, *Microsternarchus* cf. *bilineatus* Lineage C, and *Steatogenys* cf. *elegans* during conspecific playback experiments. *Microsternarchus* also exhibits two behaviors that include chirp-like extreme frequency modulations, EOD interruptions with hushing silence and tumultuous rises, and these are described in terms of receiver impact. These behaviors all have substantial impact on interference caused by conspecifics and may be a component of the JAR in some species. Chirps are widely used in electronic communications systems, sonar, and other man-made active sensing systems. The brevity of the chirp, and the phase disruption it causes, makes chirps effective as attention-grabbing or readiness signals. This conforms to the varied assigned functions across gymnotiforms, including pre-combat aggressive or submissive signals or during courtship and mating. The specific behavioral contexts of chirp expression vary across species, but the physical structure of the chirp makes it extremely salient to conspecifics. Chirps may be expected in a wide range of behavioral contexts where their function depends on being noticeable and salient. Further, in pulse gymnotiforms, the chirp is well structured to comprise a robust jamming signal to a conspecific receiver if specifically timed to the receiver’s EOD cycle. *Microsternarchus* and *Steatogenys* exploit this feature and include chirps in dynamic jamming avoidance behaviors. This may be an evolutionary re-use of a circuitry for a specific signal in another context.

## Introduction

Gymnotiform fishes of South America produce rhythmic electric organ discharges (EODs) and can detect these fields with a specialized cutaneous electrosensory system (Zupanc and Bullock, 2005). The electric fields they generate are used in two ways: As a modality of intra-specific communication important for territoriality, courtship, and mating; and as the carrier signal for an active electrosensory system capable of imaging nearby objects (Caputi et al., 2002; Caputi and Budelli, 2006; Moller, 2006). These two functions, electrocommunication and electrolocation, may not be simultaneously compatible. The distortions to the carrier field used for electrolocation can be masked or otherwise degraded by the field of a nearby conspecific, a phenomenon known as jamming (Heiligenberg, 1986). Gymnotiforms first rose to prominence as an important model system because some species possess a stereotyped jamming avoidance response (JAR). Watenabe and Takeda (1963) and Bullock et al. (1972) described the JAR of *Eigenmannia* as a response to the presence of a nearby conspecific wherein the responding fish alters the rate of their ongoing EODs to minimize the impairment created by the conspecific’s discharge. This reliable and experimentally tractable behavior has been used to determine and understand the circuitry of electrosensory analysis and the premotor and control circuits responsible for the resultant changes in ongoing EOD rate (Metzner, 1999; Rose, 2004).

In addition to the JAR, a wide range of patterned changes in EOD rate have been observed across gymnotiform species (Carlson, 2006). These electromotor behaviors are used as signals in a wide variety of communicative contexts, i.e., as adaptive stereotypic displays with specific signal functions. The best studied of these signals is the chirp, a very large transitory increase in frequency (Hopkins, 1974; Hagedorn, 1986, 1988; Dye, 1988; Shumway and Zelick, 1988; Kawasaki and Heiligenberg, 1989). The earliest observations of chirps were recorded during aggressive encounters (Bullock, 1969), but subsequent observations of chirps have shown them to occur also in reproductive and spawning behaviors as well (Hagedorn and Heiligenberg, 1985; Hagedorn, 1988). A large literature now contains a wealth of information and signal structure and functions in the gymnotiform family Apteronotidea (see Turner et al., 2007 for a systematic review), but there have been fewer studies in pulse-discharging gymnotiforms. Westby (1975) showed that the chirp is a signal mainly given by dominant *Gymnotus carapo*, and appears to often presage further aggression and can elicit submissive responses from a receiver. The more recent work on pulse gymnotiforms has elegantly demonstrated that there are specific functions for several structurally distinct chirp types in agonistic and territorial conflicts in *Gymnotus omarum* (Batista et al., 2012), and during reproductive courtship and male-male aggression in *Brachyhypopomus gauderio* (Perrone et al., 2009). This is consistent with the much larger literature on chirps in wave-type gymnotiforms, which documents a great deal of species variation in stereotyped chirp structure and their specific functions in intraspecific communication (Smith, 2013).

There is a deep phylogenetic and systematic split of gymnotiform fishes reflecting two modes of operation of the electric organ and electrosensory system (Zupanc and Bullock, 2005). The families Eigenmanniidae, Apteronotidae, and Sternopygidae are wave-type fish, with EODs that consist of roughly sinusoidal voltage modulations that are equal in duration to the length of the pacemaker cycle, creating a continuous discharge. The other gymnotiform families, Electrophoridae, Gymnotidae, Hypopomidae, and Rhamphichthyidae, are pulse-type fishes, with an EOD much shorter than the duration of the pacemaker cycle. There is great diversity across both wave and pulse type families (Crampton and Albert, 2006). In the Rhamphichthyidae, which includes the genus *Steatogenys*, EODs are generally short, around 1–2 ms, with very stable resting EOD rates between 40 and 80 Hz, although individual species typical rates may be as low as 20 Hz or as high as 120 Hz. The genera *Brachyhypopomus* and *Microsternarchus* are within the Hypopomidae, which also contains a wider diversity of EOD durations and pacemaker frequencies. The two species reported herein (*Microsternarchus bilineatus* lineage C (Maia and Alves-Gomes, 2012) and *Brachyhypopomus* sp. cf. *sullivani*) have EOD durations of ∼2–3 ms, with pacemaker frequencies between ca. 20–40 Hz in this species of *Brachyhypopomus* and 40–80 Hz in this lineage of *Microsternarchus*.

Since the majority of the EOD period is silent in pulse species, jamming interference by conspecifics is most detrimental when EODs coincide. Pulse species therefore modulate both the frequency and the relative timing of their EODs to minimize interference (Heiligenberg et al., 1978). Indeed, a major component of the pulse-type JAR is a brief rate increase that begins just before expected coincidence with the partner, resulting in fewer near-coincident EODs (Heiligenberg, 1980). Although evidence is limited to a few species, detection of other nearby objects is most impaired when a conspecific signal is presented immediately before or coincident with the animal’s own EOD (Heiligenberg, 1974; Schuster, 2002). Conspecific EODs presented immediately after the animal’s EOD have very little effect on electrolocation and this immunity to jamming persists through most of the silent inter-pulse interval (IPI). As conspecific EODs approach the later phases of the EOD cycle (just prior to the next EOD), the jamming interference again increases. This means that the most effective JAR behaviors minimize the time spent with detrimental phase relations. Effective JAR behaviors also likely depend on the ongoing pattern of phase precession to predict or judge the best time for a rate increase (Heiligenberg, 1980). This patterned phasic sensitivity to interference results (at least in part) from phasic changes in electrosensitivity (Westby, 1975; Castelló et al., 1998; Nogueira et al., 2006). The electrosensory system is most sensitive at the time of the animals’ own EOD, with a sharp decline in sensitivity immediately afterward and throughout the middle of the cycle. Sensitivity gradually recovers to a peak levels a short time prior to the next EOD. This is congruent with the observation that the time immediately prior to the EOD and perhaps briefly afterward is the most impacted by jamming interference (Heiligenberg, 1974). Conversely, the period of time after the EOD and continuing into the middle of the cycle is the least sensitive epoch and therefore most immune to jamming.

Electromotor behaviors that alter the phase precession immediately prior to or after a conspecific EOD are behaviors that manipulate jamming interference. This could also include specific signals (such as chirps) that are timed to maximize (or minimize) jamming interference or detectability based on phasic changes in receiver sensitivity. The exact duration of this sensitive time window is likely to be species specific, but we propose that discharges that occur within a 120° window surrounding coincidence are highly salient events for one or both members of a dyad. Prolonged periods of discharges within the window constitute jamming interferences. For a 50 Hz fish, 120° is equivalent to 6.7 ms. Events within the 60° window prior to a fish’s own EOD are the most salient or potentially interfering and a time window beginning shortly after the EOD is the epoch most immune to interference. In a dyad, this is clearly reciprocal, fish A discharging just before fish B (in its most sensitive window) obviously means that fish B discharged immediately after fish A (in its least sensitive window). If this phase relationship were maintained over time, we would infer that Fish A is “jamming” fish B. Electromotor behaviors that minimize discharges in that time window can be interpreted as part of a JAR, while those that increase repeated discharges within the partner’s most sensitive window may be active jamming maneuvers.

This phasic variation in sensitivity are important for understanding the impact of chirps on the receiver. The abrupt nature of chirps has large impact on phase precession between two fish. The immediate effect is a sharp change in the rather orderly phase precession between two close frequency partners. Regardless of chirp structure, this is likely to be very salient to the receiver, particularly if it results in the skipping of expected coincidences or consecutive EODs within the sensitive window. Further, if chirps result in EODs within the sensitive window of the receiver, this is likely to increase their salience.

In a series of studies on jamming avoidance behaviors, we observed chirps in *M. bilineatus* (lineage C) and in an undescribed species of *Steatogenys* (cf. *elegans*) obtained from the pet trade. This species of *Steatogenys* was unusual in that it occasionally exhibited small chirp-like behaviors spontaneously during isolated free exploration and more frequently during experiments where we presented synthetic conspecific EOD recordings. *Steatogenys* and *Microsternarchus* also showed clear evidence of chirps timed to specific phases of the stimulus cycle, and we present these in the context of putative jamming interactions. The present report describes the structure of chirps observed during conspecific playbacks in these two species as well as three individuals of a high-frequency species of *Brachyhypopomus* (cf. *sullivani)* from Amazonas state, Brazil. We describe the range of chirp structure observed, focusing especially on the resulting effects these chirps had in terms of the phase relationship with the synthetic conspecific partner.

## Materials and Methods

The subjects used in this study were hand collected in Amazonas state, Brazil, or imported through the pet trade in the United States, using exporters from Iquitos, Peru. Two different groups of *Steatogenys* were acquired through the pet trade, using the same US importers and Peruvian exporters, and were presumed to have been collected in similar localities. The first group was used in the experiments with varying playback frequencies and the second group was used in another experiment where stimulus EOD duration was manipulated. Subjects purchased from exporters were allowed to acclimate in group tanks at Hunter College for at least 2 weeks prior to testing. Subjects were also captured by hand net along the margins and banks of the Rio Negro and its tributaries in accordance with Brazilian laws and under ICMBIO permit #14833-1 to JA-G. Following capture, subjects were transported to the Laboratory of Behavioral Physiology and Evolution (LFCE) at INPA. These subjects were also maintained in group housing and tested between 2 and 4 weeks following capture. Sex and other aspects of physical condition are summarized in Table 1. All experiments were conducted in accordance with the Institutional Animal Care and Use Committee of Hunter College, CUNY, and the Ethical Committee for Animal Research of INPA.

**TABLE 1:** Subject data.

**Subject**	**Sex**	**Resting *F* in Hz (SE)**	**Length (cm)**
Steat_012	M	56.11(0.08)	18.0
Steat_014	M	49.16(.09)	17.5
Steat_015	F	55.59(0.25)	17.0
Steat_003	NA	63.73(0.24)	17.0
Steat_029	F	57.48(0.09)	19.0
Steat_036	M	59.98(0.19)	18.5
Steat_042	F	51.41(0.16)	15.5
Steat_040	NA	53.19(0.16)	18.0
Steat_017	NA	57.37(0.28)	16.0
Steat_024	NA	62.67(0.16)	16.0
Steat_018	F	52.14(0.29)	15.0
Steat_044	F	48.18(0.28)	17.5
Steat_001	F	54.56(0.26)	17.0
Steat_011	F	65.11(0.15)	16.0
SteatYC_1	F	52.05(0.39)	21.5
SteatYC_2	M	55.86(0.59)	19.0
SteatYC_3	F	59.74(0.16)	20.0
SteatYC_4	M	57.62(0.31)	21.5
SteatYC_5	M	64.44(0.46)	18.0
SteatYC_6	M	48.72(0.09)	15.5
SteatYC_7	M	58.62(0.59)	22.6
SteatYC_8	M	66.29(0.71)	21.0

Micro_01	M	62.31(0.46)	9.7
Micro_02	F	73.57(1.06)	8.6
Micro_03	M	55.04(0.78)	7.2
Micro_05	F	44.1(0.11)	7.5
Micro_06	F	48.98(0.32)	8
Micro_07	F	55.91(0.29)	8.5
Micro_09	F	58.31(0.13)	7.5
Micro_10	M	86.4(0.34)	8
Micro_11	F	58.6(0.69)	7.6
Micro_12	M	49.44(0.3)	10.5
Micro_13	F	66.57(0.31)	12
Micro_15	M	57.19(0.24)	12.4
Micro_16	F	58.01(0.37)	11.7
Micro_17	F	57.31(0.21)	10.6
Micro_18	F	40.62(0.16)	7.5
Micro_19	F	52 9 (0.14)	11
Micro_21	F	74.1(0.29)	11
Micro_22	M	58.85(0.15)	7.5
Micro_23	F	56.44(0.1)	7
Micro_24	F	56.80(0.19)	7.5
Micro_25	M	43.05(0.11)	8
Micro_26	F	52.4(0.09)	8
Micro_27	F	54.7(0.09)	7.5
Micro_28	F	49.10(0.11)	7.5

Brachy_06	NA	28.23(0.08)	8
Brachy_08	NA	22.52(0.21)	8
Brachy_09	NA	21.46(0.13)	8
Brachy_10	NA	46.36(1.03)	7
Brachy_11	NA	27.23(0.17)	7
Brachy_12	NA	37.71(0.28)	9
Brachy_13	NA	24.56(0.17)	9.5

*Tabular presentation of subject characteristics. Resting EOD frequency was measured during inter-trial intervals of stimulation experiments (see text).*

The data presented in this report are selected from a larger database of responses to conspecific playback in pulse gymnotiform species. In this report we include only individuals that displayed a behavior known as chirping and will confine our analysis to those chirps. A fuller treatment of all behaviors exhibited in these experiments is forthcoming.

### Behavioral Testing Apparatus

Animals were placed in a small plastic mesh cylinder and suspended centrally in a rectangular glass tank (25 × 40 × 20 cm) filled with roughly 14 cm of water. Subjects generally remained motionless in the tube, but the tube was sometimes closed with cloth screening if animals did not settle immediately in the tube. The recording electrodes were 8 cm carbon rods (5 mm diameter) or five loops of silver wire around a 5 mm plastic rod, located at opposite ends of the tank in parallel to the longitudinal axis of the subject. The stimulating electrode was a dipole electrode composed of either two carbon rod nubs, approximately 5 mm each, separated by 3 cm or a pair of silver electrodes separated by one half the subject body length. This stimulating electrode pair was suspended from above the tank and could be placed above the shelter, 2–4 cm from the subject’s head. During calibration of the stimulus, the electrode was rotated to parallel the recording electrodes and the subject fish, just below or above the center of mass of the subject (usually 3–4 cm behind the tip of the nose). After calibration, the electrode was kept in the same position, but rotated to approximately 15–20 from perpendicular to reduce but not eliminate the S2 recording. The entire aquarium and electrode assembly was mounted within a grounded aluminum box or a grounded single-walled sound attenuating chamber. The signal from the recording electrodes was amplified 50–500× (CWE BMA-200 or AM Systems 3000) and digitized at 48828.125 kHz using a Signal Processor (Tucker-Davis Technologies, Aluacha, FL, United States: either RM2.1, RP2.1, or RX6) connected to a windows computer. An isolation transformer was placed between all other equipment and the stimulus electrodes. All experiments and data analyses were conducted with custom routines in Visual Design Studio (Tucker-Davis Technologies) and Matlab (Mathworks, MA, United States). The signal processing system recorded both the analog waveform of the EODs during the experiment and determined the interpulse interval (IPI) of each EOD of the subject using spike timing algorithms and a voltage trigger. For measurements reported here, we measured the EOD timings directly from the analog waveform using spike detection routines in Matlab. EOD timings were collected by the signal processing computer as a record of IPIs during the pre- and post-trial periods.

As soon as visible agitation ceased and the subject (S1) began to acclimate to their surroundings (usually within 5–10 min), a recording of the animal’s EOD was digitized to be used as the synthetic conspecific, or S2. Software settings were manually adjusted to calibrate delays and amplification of the S2 during single S2 presentations at the midpoint of the subject’s IPI. The amplitude of the S2 was adjusted to 80–100% of the subjects EOD peak-peak amplitude (recorded through the same electrodes with the same amplification). The precise timing of the S2 was adjusted while shorting the stimulus electrode circuit (preventing delivery to the tank) and monitoring the timing of the recorded S1 and synthetic S2 on an oscilloscope. Equivalent phases of the S1 and S2 were matched to within 10 μs during testing at zero latency. Finally, the circuit was re-completed and the electrode position was rotated to minimize the recorded amplitude of the S2 stimulus artifact. The subject was allowed to continue acclimation for at least 15 min after calibration.

This study describes responses that occurred during S2 playback at a fixed frequency. For each trial, the S2 frequency was set relative to the subject’s frequency at the start of the trial. This initial frequency difference (dF) ranged from −16 to +16 Hz from the subject baseline and playback lasted either 10 or 15 s.

In a subset of the *Steatogenys* individuals, the S2 EOD duration was manipulated and tested at several dFs. Subjects were exposed to six different EOD durations at each tested dF, including two EODs shorter than their own and three longer EOD lengths. Individuals of *Brachyhypopomus* were exposed to three replications of each stimulus type, and individuals of *Microsternarchus* and *Steatogenys* were tested with five replications of each stimulus type. Stimulus presentation order was randomized and included inter-trial breaks of 1–3 min.

### Baseline Measurements

All trials were conducted during the subjects’ quiescent daytime period. We did not systematically control for time of day, but most experiments were conducted in a period from 4 h after lights-on to 2 h before lights-out. This was done primarily of convenience, but also because animals are extremely active during night hours and would resist confinement and present movement artifacts. While behavioral differences may be expected during more active periods, there was no shortage of electromotor responses from these quiescent subjects. It is notable that all three species tolerate large groups and probably rest in closely packed groups during the day. We used approximately 1 min of IPI recordings from each intertrial period during all experiments to estimate baseline frequency and IPI variability measurements for all subjects at the time of testing (reported in Supplementary Table S1). For each subject, we averaged all 1 min samples.

### Data Analysis

All data analysis was performed in Matlab version 2018b (Mathworks) or R (R Core Team, 2014) using publicly available packages and custom routines. We utilized the circular statistics toolbox available from the Matlab File Exchange (Berens, 2009) and PMCMR (Pohlert, 2014) for non-parametric and repeated measurements. Data with non-normal or heterogeneous variance were ln-transformed or analyzed with non-parametric tests. Multiple pairwise comparisons were performed with Bonferroni corrections.

Prior to all measurements, we digitally removed the S2 stimulus artifact using an inverted S2 waveform and the programmed S2 timings. We then used peak detection to measure the occurrence of each S1 EOD and its latency to the preceding S2. All phase angle measures were expressed relative to the fixed S2 period. The S1 timings were also converted to instantaneous measures of S1 frequency and dF (which varied from the constant initial dF as the subject altered their own EOD rate). From the S1 frequency, we derived the instantaneous change in IPI duration for each EOD interval, expressed as a percent reduction in duration for each interval relative to the interval before (rIPI). This measure is generally quite small (<1%, see section “Results”) and is useful for locating abrupt changes in pacemaker rhythm lasting for only a few intervals, such as chirps.

### Chirp Measurements

**FIGURE 1:**
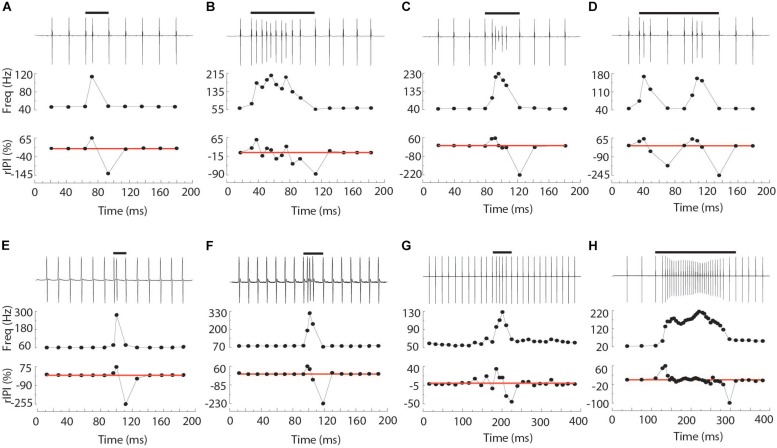
Representative chirps from *Steatogenys* **(A–D)**, *Microsternarchus* **(E,F)**, and *Brachyhypopomus* **(G,H)**. In all panels, the upper trace shows the recordings of the chirp and several preceding and subsequent intervals. The total chirp duration is marked by the horizontal black bars. The lower subpanels show the instantaneous frequency of all intervals shown and their relative reduction from baseline (IPI r) as a percentage. The thin red lines are plotted to highlight the axis position of 0% changes from baseline.

Chirps were recognized by large instantaneous increases in EOD rate. We analyzed all instances where rIPI was greater than 10%, that is all cases where the IPI decreased by more than 10% from one interval to the next (25% for *Brachyhypopomus*). For each chirp, we measured the phase angle of the first EOD in the chirp and its latency to the first delivered S2. We defined the start of the chirp as the first un-distorted EOD that begins an IPI that is at least 10% shorter than the preceding IPI. After recognition of the chirp, rIPI and amplitude reductions were measured against a baseline of the mean of three EODs prior to the chirp, or following the chirp if it occurred in the first five intervals of the trial (as was often the case in *Steatogenys*). In *Brachyhypopomus*, nearly all chirps were followed by a prolonged period of stable high frequency discharges (higher than the frequency prior to the chirp, but still much lower than frequencies achieved during the chirp). Subjects returned to a new stable frequency 20–100 ms after the last very high frequency intervals. We defined the end of the chirp as the moment when S1 frequency declined to within 10% of the post-chirp rate (measured at steady-state, at least 50 ms later). As illustrated in Figure 1A, we based our subsequent analyses on chirp duration (both number of pulses and time), maximum reduction in IPI, maximum reduction in EOD amplitude and the time to each of these latter two measures.

### Tumultuous Rise Definition

Some chirps in *Microsternarchus* occurred in the context of a large rise in baseline frequency. Algorithmically, we recognized tumultuous rises when the time-averaged coefficient of variation (CV) was greater than 5 (reflecting many chirp-like intervals) and the baseline frequency increased by more than 25%, with both conditions remaining true for more than 500 ms. The baseline frequency was averaged in windows of 20 intervals and CV was measured in blocks of 200 ms, with 50% overlap between blocks. The end of the rise was marked at the point the frequency returned to within 5 standard deviations (SD) of the pretest frequency.

### Jamming Index

To quantify the relative interference between the S1 and S2, we computed a jamming index that reflects asymmetry in the reciprocal phase relations between two fish. The jamming index was calculated by subtracting the proportion of S1 EODs that occur within 60° after the S2 from those S1 EODs occurring 60° prior to the S2 (using the total number of S1s within 120 of the S2 as the denominator in both cases). This results in an index from −1 to 1, with 0 reflecting an even distribution of S1s prior or subsequent to the S2. A jamming score of −1 indicates that all S1s in the critical window of −60° to 60° are subsequent to the S2s, meaning S1 is suffering great potential interference, while S2 is free of it. A score of 1, conversely indicates that all S1s are occurring prior to the S2, so S1 is free of interference and S2 is being jammed. We calculated jamming ratios for all trials that contained repetitive chirp-like intervals and tumultuous rises, using a sliding 400 ms measurement window, with 50% overlap between windows. To assess the statistical significance of this index, we generated 10000 uniform distributions of phase angles for matching numbers of S1 pulses in each time window. The standard deviation of the resulting shuffled jamming indices was used to convert each measured jamming index to *Z*-scores. Jamming indices with *Z*-scores greater than 1.96 were interpreted as significant measures of non-reciprocal jamming interference.

## Results

We observed chirps in a range of controlled and uncontrolled settings. The following description is based on recordings from two distinct behavioral experiments, one in which only the frequency of the stimulus fish (S2) was systematically varied, and one in which the duration of the S2 EOD was systematically varied and presented at a range of fixed S2 frequencies (dF = 0, ±0.5, ±1, ±5, ±8, ±16 Hz). All observations of *Microsternarchus* (subject *N* = 12, chirp *N* = 2555) and *Brachyhypopomus* (*N* = 3 and 58) were recorded during the S2 frequency experiments. Observations of *Steatogenys* chirps were taken from two groups of subjects, one group tested in the S2 duration experiments (subject *N* = 7) and another group tested in S2 frequency experiments (*N* = 14). In the presentation of chirps in *Steatogenys* in comparison to the other two species, the individuals from both experiments were pooled (*N* = 21; chirp *N* = 1314). Analysis of responses as a function of S2 frequency or duration, as well as other behavioral responses to conspecific stimuli, will be presented in a subsequent publication.

### Chirp Structure

Chirps are distinct from other electromotor behaviors in their abruptness. That is, chirps contain dramatic modulations that reach their maximum within a very small number of EOD intervals. We recognized chirps by the magnitude of interval-to-interval reduction in duration (rIPI). In baseline measurements (in the absence of chirps), the *Steatogenys* subjects had a mean rIPI of 0.0062%, with a standard deviation of 0.27. *Microsternarchus* subjects had a mean rIPI of 0.0022% (SD = 0.005). *Brachyhypopomus* subjects had a mean rIPI of 0.0297%, with a SD of 0.01. A large frequency shift during jamming avoidance or a startle response may have a peak rIPI of ca. 5%, with the majority of electromotor behaviors comprised of changes of less than 1 or 2% and persisting over more than 10 intervals. This is especially true for highly regular species like *Microsternarchus* and *Steatogenys*. Chirps were easily recognizable because they included extremely sudden instantaneous changes (rIPI > 10%). Large gradual frequency shifts in *Brachyhypopomus* may include rIPI values of 5–10%, so we used a higher chirp threshold of 25% rIPI in this species.

We observed chirps in a subset of individuals in all three species (S: 21 of 30 individuals tested in similar conditions; M: 12 of 24; B: 3 of 7). The three chirping individual *Brachyhypopomus* were undifferentiated and sex could not be determined. The majority of individuals were immature (stage 1 or 2), and only one male *Steatogenys* (SteatYC2) was stage 3 mature (following Crampton and Hopkins, 2005). Males, females and immature individuals of *Microsternarchus* and *Steatogenys* all chirped. We found no detectable statistical difference between sexes in numbers of chirps, chirp duration or reduction in either IPI or amplitude (Wilcoxon rank sum, all *p* > 0.05). In *Microsternarchus*, we also failed to find any significant correlation between chirp numbers, duration or modulation of IPI and amplitude with subject length (Spearman Rank, all *p* > 0.05). We had a greater range of size and maturity in *Steatogenys* and the most mature males (SteatYC2 and SteatYC7) were prolific chirpers, but there was no correlation between animal size and numbers or chirps or chirp durations in this species either. There was, however, a modest relationship between the amount of modulation within the chirp and animal size. Reduction of IPI was significantly correlated with animals size (*r*^2^ = 0.28, *p* < 0.05), as was peak frequency (*r*^2^ = 0.48, *p* < 0.01), and rAMP (*r*^2^ = 0.25, *p* < 0.05).

**FIGURE 2:**
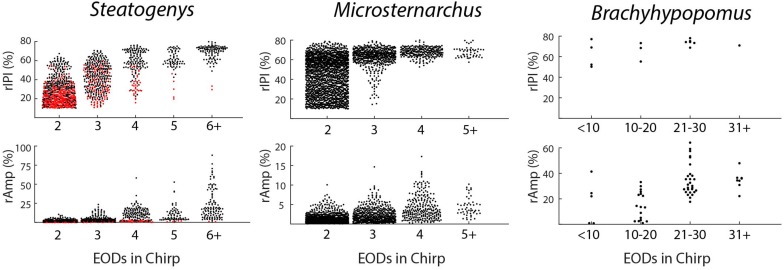
Beeswarm plots of chirp parameters for all three species. The **upper** and **lower** panels depict the distributions of maximum IPI reduction and EOD amplitude reduction, respectively, each plotted as separate distributions according to the number of EODs comprising the chirp. In the *Steatogenys* panels, red circles indicate chirps recorded from the dF experiment group and black circles indicate chirps recorded from the EOD duration experiment group.

**FIGURE 3:**
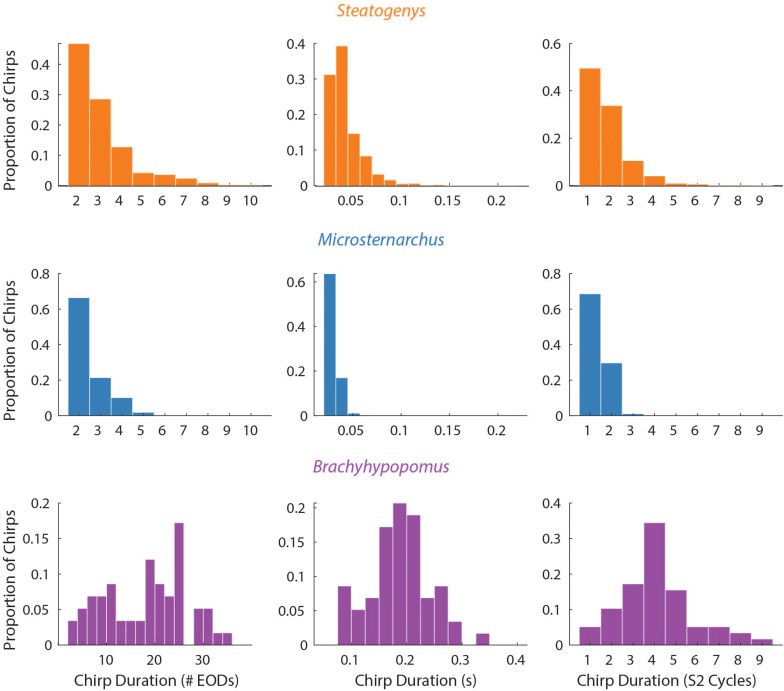
Probability histograms of all chirps (pooled) in all three species, plotted in terms of three measures of chirp duration: number of EODs (left), time (middle), and number of S2 cycles affected (right). Chirp duration expressed as S2 cycles were the only metric that allows comparison across species on the same scale.

Chirps in both *Microsternarchus* and *Steatogenys* were short, with two to six EODs comprising a chirp in *Microsternarchus* and two to 11 EODs in *Steatogenys* (Figure 1). *Microsternarchus* chirps were more stereotyped, with the majority (67.5%) comprised of a single interval shortened by an average of 44.1% (Figures 2, 3). Reduction of EOD amplitude during the chirp was generally small, with a maximum of 17.3% (Table 2). The shortened chirp intervals were immediately followed by a period of stable discharge rates similar to the preceding baseline frequency. Chirps in *Steatogenys* were also mostly short, with 45.8% comprised of a single interval shortened by an average of 27.9% from the preceding interval. As in *Microsternarchus*, shortened chirp intervals in *Steatogenys* are immediately followed by a resumption of regular discharges similar to the preceding rate (Figure 1). In the combined sample of *Steatogenys*, there was a relationship between chirp length and individual size, with the largest individuals displaying longer chirps with more complex patterns of amplitude and interval reduction (Table 2 and Figure 2). In Figure 2, which shows the relationship between the numbers of EODs in a chirp and other chirp metrics, the group of smaller individuals (S2 dF experiment) was plotted as red dots. Chirps recorded from these individuals were generally smaller in all metrics we applied, but it should be noted that all individuals displayed an overlapping range of chirp intensities. Similar plots separated by individuals are shown in Supplementary Figure S1.

**TABLE 2:** Summary statistics of chirp metrics in the three genera.

		**Chirp metrics**						
		**Mean (SD) (min–max)**						
	**# Chirps**	**Duration**						
		**EODs in chirp**	**Time (s)**	**# S2 cycles**	**Starting phase (°)**	**rlPl (%)**	**pkF (Hz)**	**rAmp (%)**
*Steatogenys*	62.571 (62.092) (1–203)	2.610 (0.718) (2–4.537)	0.038 (0.007) (0.025–0.057)	2.158 (0.317) (1.629–2.906)	214.918 (85.798) (22.254–320.040)	31.421 (14.855) (13.266–69.737)	91.636 (27.519) (68.794–159.787)	3.161 (5.241) (0.323–23.0523)
*Microsternarchus*	212.917 (358.350) (5–1232)	2.231 (0.237) (2.044–2.825)	0.031 (0.005) (0.023–0.041)	1.748 (0.181) (1.438–1.983)	233.317 (126.746) (43.336–359.853)	41.961 (9.522) (23.499–58.810)	121.195 (39.619) (67.749–224.623)	1.503 (1.380) (0.499–5.609)
*Brachyhypopomus*	19.333 (18.037) (2–38)	21.135 (1.540) (19.667–22.737)	0.193 (0.023) (0.170–0.217)	4.619 (0.670) (4.160–5.388)	150.762 (82.553) (100.903–246.052)	83.598 (4.017) (79.813–87.812)	196.370 (14.188) (187.872–212.748)	25.541 (8.924) (15.678–33.057)

*For each genus, the table provides the mean, standard deviation, and range for numbers of chirps recorded per individual, with combined averages for all other chirp metrics. The individual subject means are given in Supplementary Table S1.*

*Brachyhypopomus* chirps were longer, with at least six shortened intervals, usually with modest EOD amplitude reduction that increased gradually to a maximum near the midpoint of the chirp and gradually returned to full amplitude by the end of the chirp. Following the chirp, subjects gradually returned to a new baseline rate substantially higher than the preceding rate. IPI modulations were much greater in this species, with a mean IPI decrease of 82.3% and mean amplitude reduction of 27.5%.

The duration of each chirp is a product of the number of modulated EOD cycles, the underlying base frequency, and the degree of IPI reduction. This complicates comparison across species that differ in typical rates, as is the case for the three species herein. Since all of the chirps we analyzed were recorded during interactions with a synthetic conspecific, we translated chirp duration to a measure that reflects the perceptual experience of the chirp recipient. That is, we divided the chirp duration (in seconds), by the duration of a single S2 interval and converted the result to degrees of phase. This measure allows the comparison of chirps in units of S2 cycles (i.e., 360° per cycle), and these results are shown in Figure 3. In these terms, each chirp generally interacts with a small number of S2 cycles, from 1 or 2 in *Microsternarchus*, 1–5 *Steatogenys*, and 1–9 in *Brachyhypopomus* (although most chirps interact with fewer than five S2 cycles in this species as well).

Regardless of units, chirp durations were significantly different across individuals of these three species. A Kruskal–Wallis test found significant differences between all three species (χ^2^(2) = 69.386, *p* < 0.001), with significant pairwise differences between all pairs (*p* < 0.001). Predictably, the same result was found when this test was applied to chirp durations expressed as either S2 cycles (χ^2^(2) = 111.928, *p* < 0.001) or as EODs per chirp (χ^2^(2) = 75.364, *p* < 0.001), again with all pairwise comparisons being significantly different (*p* < 0.001).

The most prominent feature of chirps is the IPI reduction, but the magnitude differed greatly across species (χ^2^(2) = 40.44578, *p* < 0.001). *Brachyhypopomus* chirps had a greater reduction of IPI than chirps in either of the other two species (*p* < 0.001), but the distributions of rIPI were overlapping in *Microsternarchus* and *Steatogenys* and not statistically distinct. In frequency terms, however, the peak frequency (i.e., the inverse of the shortest chirp interval) did differ in all pairwise comparisons (χ^2^(2) = 44.709, *p* < 0.001: *Steatogenys* vs. *Microsternarchus p* < 0.05; *Brachyhypopomus* vs. either species *p* < 0.001).

In all three species, the individual EODs within a chirp were often reduced in amplitude (see Figures 1, 2). Median EOD amplitude reduction was greatest in *Brachyhypopomus* (27.4%), and substantially less dramatic in both *Microsternarchus* (0.89%) and *Steatogenys* (1.8%). Although the median amplitude drop was similar in these two species, there was a much greater variability of EOD reduction in *Steatogenys*, possibly reflecting a wider range of subject maturity in those subjects All pairwise comparisons of amplitude reduction were significantly different between species (χ^2^(2) = 44.670, *p* < 0.001).

All three of these aspects of chirp structure, duration (regardless of units), IPI reduction (and max frequency), and EOD amplitude reduction, are tightly interdependent (Figure 2). Both IPI reduction and amplitude reduction increased as chirps got longer, but the distribution of amplitude reductions also widened. Many long chirps had little or no amplitude reduction and some short chirps had greatly reduced EOD amplitudes (see Figures 1B,D for examples, and Figure 2).

### The Chirp and Disruption of Phase Precession

**FIGURE 4:**
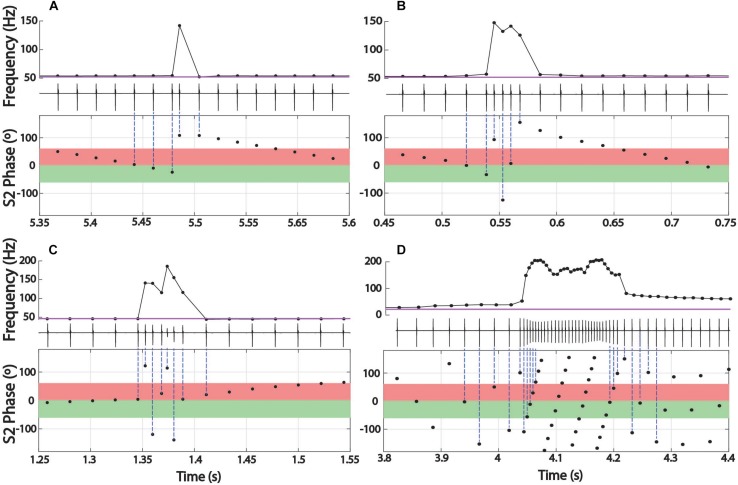
Phase precession during chirps. Three representative chirps in *Steatogenys* are shown, simple **(A)**, long **(B)**, and complex **(C)**. A representative chirp from *Brachyhypomous* is shown in **D**. In each panel, the top subplot shows the instantaneous frequency, with the oscillogram trace directly below. The bottom subplot shows the phase of each S1 EOD with respect to the stimulus (S2). Each EOD is represented by a black circle, with dotted lines connecting to the chirp EODs for reference. The S2 frequency is depicted in the frequency plots by the horizontal purple line. In the phase plots, the 60° window before and after the S2 EODs is indicated by green and red boxes, respectively.

The interference pattern of two individuals with closely matched frequencies consists of a stable precession of phase relationships, particularly in gymnotiforms with very regular rates, like *Microsternarchus* and *Steatogenys*. The large abrupt changes of chirps created particularly prominent breaks in phase precession (Figure 4). This was especially true of longer chirps, during which each successive EOD in the chirp occurred at scattered (and presumably unpredictable) phases across multiple S2 cycles. In *Microsternarchus*, with the shortest and most stereotyped chirps, the break in phase precession was the most prominent effect of the chirp. In *Steatogenys* chirps, with some variability of IPI within a chirp, this resulted in a scattering of S1 EODs across a few S2 cycles (Figures 4B,C). In *Brachyhypopomus*, which had more consistent rates during the chirp, the phase precession during the chirp appears regularized, reflecting a typical precession between partners with a large frequency difference (Figure 4D). This also had the effect of increasing the number of consecutive EODs within the partner’s sensitive jamming window.

### Chirp Occurrence Patterns and Timings Relative to the S2

**FIGURE 5:**
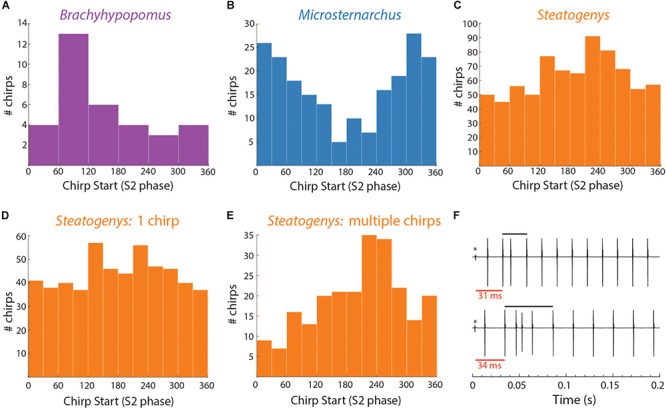
Distributions of chirp starts in terms of S2 phase **(A–E)** and examples of early latency chirps in *Steatogenys* **(F)**. **(A–C)** Overall distribution of mean chirp starting phases for *Brachyhypopomus*, *Microsternarchus*, and *Steatogenys*. **(D,E)** The distribution of chirp phase angles in *Steatogenys* subdivided by trials that contain only a single chirp **(D)** vs. the distribution of chirp phase angles in trials where there is more than one chirp **(E)**, showing a significant concentration of phase angles around 240°. **(F)** Examples of short latency chirps in *Steatogenys*. For illustrative purposes, the first S2 stimulus artifact was not digitally removed from these records (^∗^). The red bars show the latency from the first S2 to the start of the first chirp intervals. Black bars indicate the total chirp duration.

In *Brachyhypopomus*, chirps occurred as single events (sometimes multiple times in a single trial) or in groups with an extended period of high frequency baseline between chirps. Of 58 chirps observed, 18 (31%) occurred as single events in a trial. The remainder of chirp observations occurred in trials with more than one chirp, with an average of 2.5 chirps per trial (0.17 chirps/second). Chirps did not occur uniformly throughout the trials, but rather in clusters of 2–6 chirps, with an average spacing of 2.7 s between chirps. In trials with several chirps, they often occurred at consistent phase angles throughout the trial (the mean SD of phase angles within a trial was 62.5°). The distribution of mean phase angles in these 34 trials is shown in Figure 5.

This distribution had a mean starting angle of 104.5° (SD = 93.6°), but could not be distinguished from a null hypothesis of uniform distribution (Rayleigh test *r* = 0.26, *p* > 0.05).

**FIGURE 6:**
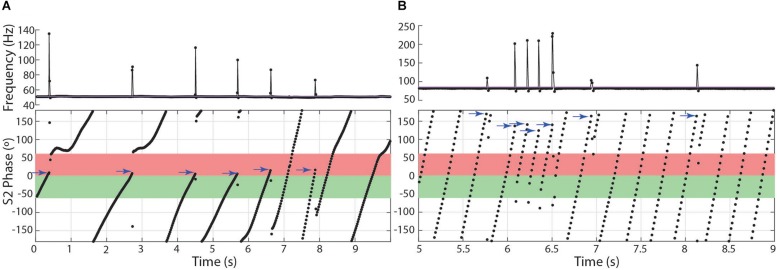
Patterns of chirp occurrence during stimulus trials. **(A)** *Steatogenys*, **(B)** *Microsternarchus*. In trials with multiple chirps, successive chirps often occur at consistent phase angles (blue arrows).

In the other two species, subjects chirped in a number of distinct patterns through the 10 or 15 s stimulus presentations. In both *Microsternarchus* and *Steatogenys*, it was common for a trial to contain multiple repetitive chirps with inter-chirp intervals roughly equal to the S1–S2 beat cycle duration. In both species, chirps often occurred once per beat cycle, with consistent phase timing for each chirp (Figure 6). The specific phase angle of chirps within a trial differed between trials, and varied from moment to moment within a trial, but repeated chirps often occurred at consistent phase angles for each beat cycle for a periods of 1–3 s of these trials. The duration of the beat cycle is the inverse of the frequency difference between the two signals, so inter-chirp intervals were dynamically matched to the frequency difference between the two signals, despite variation in S2 frequencies across trials.

In *Microsternarchus*, chirps nearly always occurred in multiples. Of 2555 chirps observed, only 34 (0.01%) occurred as single events in a 15 s stimulus presentation. Multiple chirps occurred in 169 stimulus trials, with a mean of 14.9 chirps per trial (range = 2–118). In many instances, chirps occurred at consistent phase angles, with chirps at the same phase of every beat cycle (Figure 6B) for periods of several seconds (mean SD within trials was 64.7°). The distribution of mean phase angles per trial (Figure 5) was significantly concentrated around a mean vector 358.4° (SD = 92.9°, Rayleigh test *r* = 0.27, *p* < 0.001), or just prior to the next S2. The dispersion indicates that the mean phase angles of most trials clustered either immediately before or after the S2.

The number of chirps in individual trials was too low to apply inferential statistics, but we analyzed the distribution of chirp angles for each individual, pooled across all trials. Six individual *Microsternarchus* had a significant concentration of phase angles (*p* < 0.05) and these distributions are shown in Supplementary Figure S2. The concentration of these distributions was weak (*r* < 0.41), but show a general tendency to avoid mid-cycle chirps, with a mean of 341.5° (SD 44.6) (Supplementary Figure S2).

**FIGURE 7:**
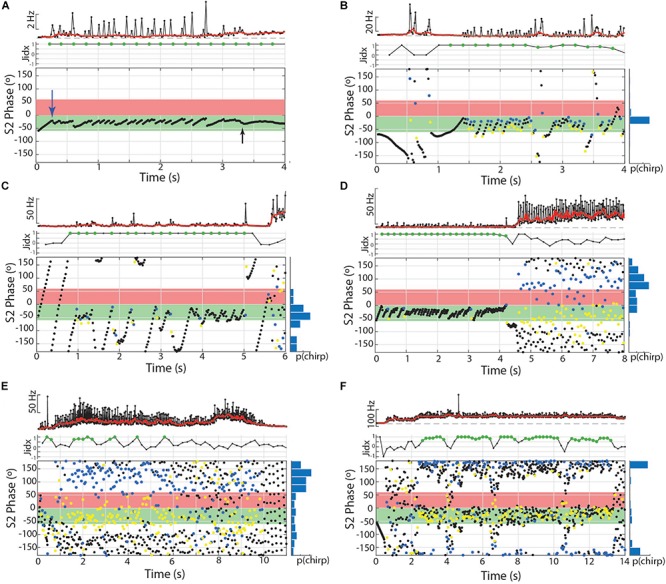
Chirps and chirp-like intervals manipulate phase angle during burst-like chirping and tumultuous rises in *Microsternarchus*. **(A)** Subthreshold chirp-like intervals are timed to occur repeatedly at S2 phase angles very late in the S2 cycle (the first one is indicated by the blue arrow) at approximately –5°, equal to 355°. At 2.75 s, the chirp-like intervals are no longer expressed, but the subject switched to a steady matched frequency with a consistent phase of ∼–10° (black arrow). In this and subsequent panels, the jamming index is plotted, with significant positive values shown as green circles. **(B)** Burst-like chirping (these spikes are well above chirp-threshold of 10% rIPI) produced chirps every 2–6 intervals to maintain jamming avoidance and to fill the critical window of the S2 with EODs. In this and subsequent panels, the normalized chirp probability is shown as a histogram to the right of the phase plot. In all phase plots, the blue circles indicate the start of each chirp and yellow circles mark the location of the next EOD (within the chirp). **(C)** Another exemplary burst-like chirp episode. In this case, the timing between chirps was matched to a multplie of the S2 frequency, resulting in fewer, more widely spaced chirps, particularly during seconds 1–3. During this period, the S1 avoids being jammed but does not fill the critical phases of the S2 with EODs as occurred with the shorter, more frequent chirps in **(B)**. **(D)** Jamming by burst-like chirping followed by a tumultuous rise that does not include well matched inter-chirp timings. In the final 4 s of this example, there is no specific asymmetry of jamming interactions, but S1 EODs mostly avoid the critical windows. **(E)** Example of a tumultuous rise that produced some asymettric jamming but with less specificity than in **(D)** or **(F)**. **(F)** Example of a tightly matched tumultuous rise resulting in nearly 12 s of jamming the S2.

Several individual *Microsternarchus* also produced chirps in rapid succession, with multiple chirp-like intervals per S2 cycle separated by only a few “normal” intervals between chirps (Figure 7B). This occurred with sometimes with minimal change to baseline frequency or with large increases. These events will be described below, as burst-chirping and tumultuous rises, respectively. *Steatogenys* did not display burst-like repetitive chirping, although some long chirps could be interpreted as multiple chirps with very little time separation between them (e.g., Figure 1D) or possibly as an immature example of a longer chirp.

Aside from the absence of rapid repetitive chirping, *Steatogenys* chirping behavior was similar to *Microsternarchus* in that multiple chirps in a single trial were matched to the beat frequency and frequently occurred at similar phase angles (mean SD of individual trials was 52.2°). Of the 1314 chirps included in this analysis, 529 (40.2%) occurred as a single chirp in a 10 s stimulus presentation. The remaining 59.8% of chirps occurred as repeated events within a single trial (232 trials). These trials contained a mean of 3.4 chirps per trial (range: 2–11). The mean starting phase of chirps across trials in *Steatogenys* was directed around a mid-cycle mean of 218.4° (SD = 117.0°), but the concentration was weak (Rayleigh test *r* = 0.12, *p* < 0.001). In trials that only contained a single chirp (Figure 5D), the distribution of phase angles was uniform (*r* = 0.07, *p* > 0.05). For trials that contained multiple chirps, however, the mean start phases were more narrowly directed around a mean of 229.5° (SD = 93.6°, *r* = 0.26, *p* < 0.001, Figure 5E), or slightly past the mid-point of the S2 cycle. This tendency was also apparent in the chirp angles pooled across trials for each individual (Supplementary Figure S2). Fifteen individual *Steatogenys* (of 21 that chirped) exhibited a significant concentration of phase angles across trials (Supplementary Figure S2). Unlike the late-cycle chirps of *Microsternarchus*, most *Steatogenys* subjects chirped closer to mid-cycle, with a mean concentration of these chirps were concentrated just after the mid-point of the cycle, with a mean S2 angle of 233.2° (SD = 49.8°).

We also noticed that many *Steatogenys* trials included a short latency chirp, very quickly following the first S2 presentation. Of trials that contained chirps, 22.8% had a chirp within 100 ms following the first S2 (mean latency = 49.7 ms). In many cases, the chirp occurred immediately, with only one “normal” interval between the first S2 and the start of the chirp. Two examples of very short latency chirps are shown in Figure 5F). This was not observed in the other two species. Behavioral responses did occasionally occur within the first 100 ms in the other two species, but these were infrequent and there were none that occurred earlier than 50 ms after the first S2.

*Steatogenys* was also unique among the species studied in that we observed isolated individuals chirp without an apparent eliciting stimulus. We have observed some individuals chirping dozens of times per 24H of EOD recordings in isolation. Further, all individuals that displayed chirps did so within the first 100 ms in at least one trial, and six individuals chirped within the first 100 ms on more than 10% of trials, with a range of 2–80% of trials containing short latency chirps (mean = 14.7%, SD = 22) across individuals. Kruskal–Wallis analysis of the minimum latencies observed in these three species revealed a significant difference (χ^2^(2) = 25.715, *p* < 0.001), with significant pairwise differences between *Steatogenys* and both of the other species, but no significant difference found between *Microsternarchus* and *Brachyhypopomus*.

### Other Behaviors With Large Instantaneous Interval Reductions: Burst-Like Chirping and Tumultuous Rises

**FIGURE 8:**
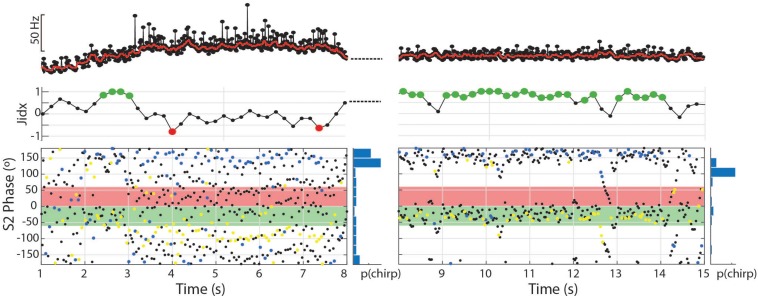
The dynamic nature of the tumultuous rise allows subjects to find matching chirp intervals and jam the S2. In the first 7 s depicted, 81 chirps occurred with a mean phase angle of 160.8° (*r* = 0.49, *p* < 0.01). In the second 7 s depicted, the subject shifts to a stable frequency and continues to produce chirps timed to the S2 period, but fewer and with greater consistency (56 chirps, mean = 168.5°, *r* = 0.72, *p* < 0.01).

We observed two other electromotor behavior patterns in *Microsternarchus* that contain the abrupt changes in IPI and EOD distortion seen in chirps: burst-like chirping and tumultuous rises. Twelve individuals (nine males and three females) displayed burst-like repetitive chirping, with chirp-like intervals (i.e., >∼10% shorter than baseline) repeated almost continuously with fewer than three “normal” intervals between them (Figures 7, 8). These events ranged from 0.63 to 12.9 s, with a mean duration of 5.9 s (SD 4.3). Burst-like chirping episodes contained from seven to 123 chirp-like intervals for an average of 34.0 chirp-like intervals per episode, or 5.8 chirps per second (range: 0.16–17.9 chirps per second). Examples of burst-like chirping are shown in Figures 7B–D.

**FIGURE 9:**
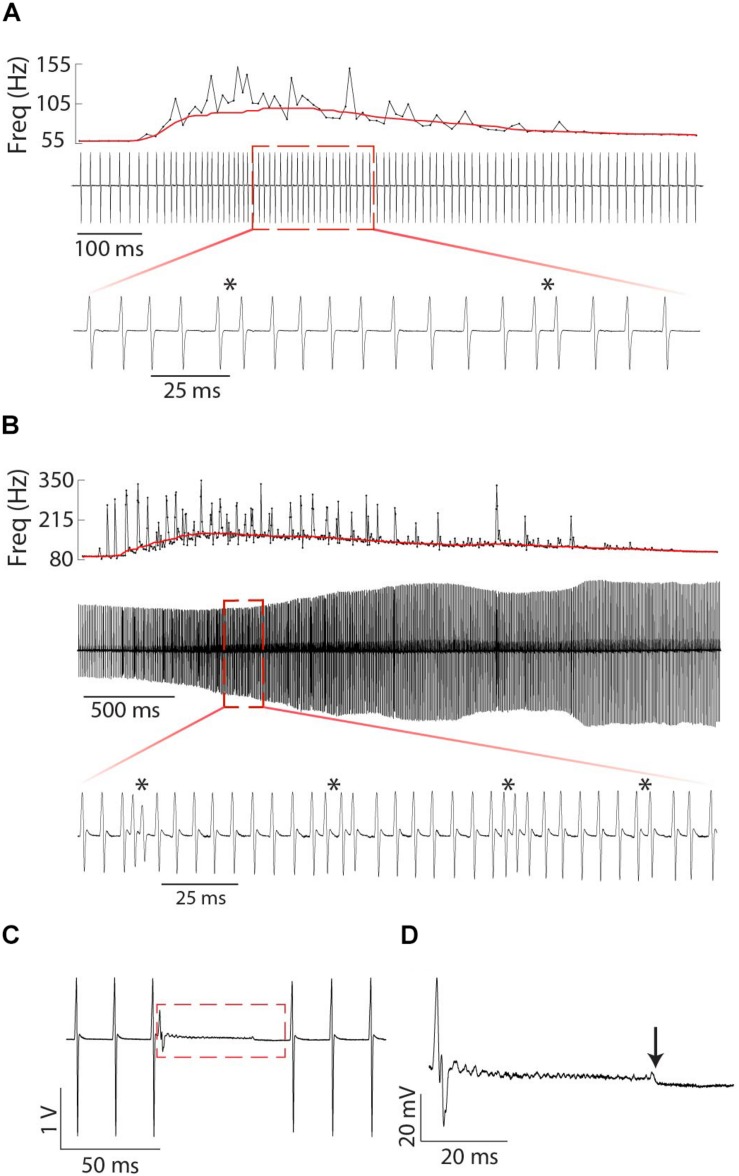
Fine structure of tumultuous rises and interruptions in *Microsternarchus*: Relatively small **(A)** and large **(B)** Tumultuous Rises. In each panel, the top plot shows the instantaneous frequency of every EOD during the entire duration of the tumultuous rise, with a solid red line depicting the ongoing baseline after filtering with a moving window of 250 ms. The middle plot of each panel shows an oscillogram of EODs during the same time period. The expanded bottom plots show a smaller segment of time to illustrate individual chirp-like events, marked by asterisks. The interruption in **(C)** occurs abruptly, with no change to the ongoing EOD frequency. There is some apparent recovery in EOD amplitude following the interruption, but the frequency is unchanged. **(D)** Expanded view of the red rectangle in **(C)**. Interruptions always began with a very shortened IPI and a greatly reduced EOD, followed by a noisy hash. The hash typically ends midway through the interruption (downward arrow). Voltage and time scales for both panels illustrate scale directly. Note that the amplitude modulation of **(B)** is caused by animal motion relative to the electrodes.

The same twelve individuals, as well as two others (10 females and 4 males) also exhibited a repetitive chirping behavior paired with a very large increase in baseline frequency (Figures 7D–F, 8, 9A,B). We term this behavior tumultuous rise due to its similarity to the tumultuous rise described by Kawasaki and Heiligenberg (1989). Tumultuous rise events lasted from 0.68 to 15.78 s, with a mean duration of 6.65 s (SD 3.6). The average frequency rise from baseline was 34.3 Hz (SD 18.73), measured at the midpoint of the rise. The peak frequency of the baseline during the rise was an average of 62.54 Hz (SD 32.77) above the frequency prior to the rise (max = 187.73 Hz). Throughout the frequency rise, subjects exhibited closely packed chirp-like intervals. Individual tumultuous rise events contained between five and 245 chirp-like intervals for an average of 99.34 (SD 56.42) chirps per rise, or 15.61 chirps per second during rises (SD 6.23).

Tumultuous rises occurred more often when the starting dF was negative (167 instances where the initial S2 frequency was lower, vs. 78 instances with a higher starting S2: Mann–Whitney *U* = 1182.5, *p* < 0.001), but it should be noted that the large frequency rise resulted in negative dFs (S2 lower frequency) during all tumultuous rises.

Both bouts of burst-like chirping and tumultuous rises often contained periodic structure that resulted in consistent placement of S1 EODs across the S2 cycle (Figures 7, 8). In some cases, repeated chirps at high phase angles (just prior to the S2) resulted in consecutive runs of closely spaced EODs within the sensitive window of the S2 (Figures 7B,D). Larger and less frequent chirps resulted in successive short blocks of interference separated by periods of neutral phase relations (as in Figure 7C). A similar pattern also occurred in the absence of chirps, but with repeated interspersed short intervals below the 10% rIPI chirp threshold, as in Figure 7A. Thus it is possible that there is a continuum of behaviors from jamming maintained by burst-like chirp intervals to smooth jamming by fixed frequency matching (as in the last ∼1.25 s of Figure 7A). Unlike these repetitive chirp behaviors, smooth jamming by matched frequency was generally infrequent, but was seen in at least one trial for 7 of 24 individuals tested.

During tumultuous rises, the high frequency of the S1 resulted in many more EODs distributed throughout the S2 cycle. If the fish interspersed chirp-like intervals specifically timed to the S2, the S1 EODs could be concentrated at specific phase angles. That is, with a fixed S2, if the time between chirps is equal to the S2 duration, or some whole multiple of it, the chirps will occur at consistent phase angles. The timing of EODs within the chirps will depend on chirp structure and the difference in frequency from the S2. In most trials, the phase of chirp-like intervals was roughly consistent within a bout, and coordinated with the harmonic relationship between S1 and S2 such that a majority S1 EODs occurred within specific windows of the S2 cycle. These windows occurred at a range of values, with the first EOD per S2 cycle occurring before, simultaneously with, or after the S2. This appeared to be a dynamic process where subjects adjusted their own base frequency, the timing and consistency of chirps, and perhaps the chirp structure to match the stimulus. This is illustrated in Figure 8. In this example, as the subject began a tumultuous rise, it briefly passed through a dF that aligned the chirps and resulting EODs to the S2 cycle (between second 2 and 3). As the frequency increased further, the alignment of S1s to the S2 cycle degrades. Although the inter-chirp timing was matched to the S2 (as can be seen in the phase histogram), the duration of S1 intervals, both within the chirps and between them, was not coordinated and the subsequent EODs within and following each chirp arrived at a range of S2 phases as the S1 frequency shifted. At the 8th second, the fish abruptly shifted its frequency downward and regularized its interchirp timing, producing chirps with nearly every S2 cycle. This pattern was sustained for the next 6 s.

To examine the matching of chirp timing to the S2, we applied the Rayleigh test to each individual trial containing tumultuous rises. Of the 210 trials that contained tumultuous rises or burst-like chirping, 144 had significantly directed concentrations of chirp phase angles (mean angle = 227.8°, SD 51.3). For individual animals, the percentage of phase-locked trials ranged from 25 to 93% (one subject produced only two tumultuous rises, and both were significantly phase locked). Most of these events resulted in the pattern shown in 7F, with a high concentration of S1 EODs placed immediately prior to the S2 and resulting in clusters of S1 EODs within the critical window of S2, with a very small number falling in the S2 phases most detrimental to the S1.

To further characterize the effect of this behavior on jamming interactions in a dyad, we calculated a jamming ratio in 400 ms windows throughout these trials. The jamming ratio is a ratio of the number of EODs prior to the S2 relative to the number after the S2, with scores close to +1 indicating jamming of the S2 and scores close to −1 reflecting jamming of the S1. For these individuals, 12.5–74.1% of trials analyzed contained significant periods of jamming, with a positive jamming index indicating that the S1 was actively jamming the S2. Periods of significant jamming indices ranged from 400 ms to 6.4 s. Epochs with significantly negative jamming indices were rare in comparison, occurring only 14 times in 11 trials from seven of these individuals. The longest of these negative jamming epochs was 800 ms and most lasted only 400 ms (a single measurement window).

### Interruptions

In three individual female *Microsternarchus*, we observed 22 interruptions in EOD rhythm lasting from 39.5 to 91.1 ms. This behavior began with a sudden EOD deformation and massive frequency increase, typically followed by a period of high-frequency hash. The high-frequency hash generally persisted for only a portion of the silent period, followed by a low-noise pause before an abrupt return to baseline EOD production (Figure 9C). All three individuals displayed interruptions during both negative and positive S2 stimulus presentations, although more interruptions occurred when the S2 stimulus was higher frequency at the start of the trial.

## Discussion

We found that three species of high-frequency pulse gymnotiforms produce chirp-like modulations of their EOD frequency that could function as communication signals or as jamming avoidance behaviors, or both. In our laboratories, we have conducted similar experiments and had similar opportunities to observe chirps in seven other genera (including more than ten species), but we only observed chirps in these particular species of *Steatogenys*, *Microsternarchus*, and *Brachyhypopomus*. In our samples of *Microsternarchus* and *Brachyhypomus*, chirping was individual specific and possibly unusual. In *Brachyhypomus*, only three individuals produced chirps in these experiments. In *Microsternarchus*, a majority of individuals tested here chirped, but we have also examined several closely related species without ever observing chirps. In all three species studied here, there was great individual variation in chirp proclivity, with some fish chirping only a few times and others chirping hundreds of times over the course of a 1 or 2 h experiment.

Among pulse fishes, this species of *Steatogenys* is unusual for its frequent use of chirps. We have observed chirps in casual observation of fish housed in solitary tanks, with estimates of spontaneous chirping in these individuals ranging from 1 to 15 chirps per 24 h of observation (Field, 2016). Chirps have been observed in males and females and become common in individuals larger than 15 cm of either sex. In many of the trials reported here, the chirp appears similar to a startle or orienting response in its latency and repeatability, especially when presented with higher absolute dF stimuli. This possibility requires continued investigation, but it is also clear that chirps are a common feature of jamming interactions and are most often observed in interactions with low absolute dF stimuli. This is also true of our observations of chirping in *Brachyhypopmus* and *Microsternarchus*.

### Sex and Maturity of the Individuals Observed

The present findings are constrained by the lack of diversity in our sample. Several individuals were too immature to assign to a sex. None of our subjects were in breeding condition and the most mature fish in our sample were only stage 2 or 3 on Crampton and Hopkins (2005) scale of maturity (=maturing-mature). This does not change the finding that *Steatogenys* chirps frequently and that all three species sometimes chirp when presented with conspecific stimuli, but it does limit our interpretations of these findings. We cannot yet comment on the specific behavioral context for chirp expression (e.g., reproductive, agonistic) or any specific correlations between chirp structure and behavioral usage.

### Comparison of Chirps Presented Here and Those of Other Species in the Literature

The chirps we reported for *Brachyhypopomus* sp. are similar to those described for other *Brachyhypopomus* species, with perhaps the greatest similarity to type-M as described by Perrone et al. (2009). The type-M chirp was only observed in male-male interactions, but our subjects were not mature enough to be differentiated. Kawasaki and Heiligenberg (1989) distinguished “strong” and “weak” chirps in *Brachyhypopomus brevirostris*, but the analysis of Perrone et al. (2009) suggests that there may be several subtypes of chirps subsumed by Kawaski and Heiligenberg’s title of “strong” chirps. The type-M and the chirp reported here are both somewhat intermediate between strong and weak, in that they are longer and have greater modulations than weak chirps, but have little deformation of the EOD waveform within the chirp, other than a 20–30% reduction in amplitude.

The weak chirps in Kawasaki and Heiligenberg (1989) and the decrement bursts of *Brachyhypopomus occidentalis* described by Hagedorn (1988) are very interesting in relation to the chirps seen in *Microsternarchus* and *Steatogenys*. In all cases, the modulations were short, consisting of a small number of intervals. In *B. brevirostris* and *B. occidentalis*, and in *Microsternarcus*, weak chirps were reported in sequences of many interspersed chirps or decrement bursts. Kawasaki and Heiligenberg (1989) did not report on the phase relations with the receiver, but it is possible that the weak chirps they observed were timed to specific phase relations, as the chirps were in *Microsternachus*. The tumultuous rise reported in that paper differed from that in *Microsternarchus* defined here, in that *B. brevirostris* did not intersperse chirps in the tumultuous rise, although they do report “frequency-dependent decrements in EOD pulse amplitude” (p. 734) and their examples come from freely moving individuals, complicating detection. These authors only detected tumultuous rises during courtship interactions.

### Chirps as Signals vs. Chirps as Phase Manipulation for Jamming Avoidance

In the existing literature, chirps have been treated as signals, with specific structure presumably selected for specific responses from a receiver (e.g., Perrone et al., 2009). It is possible that at least some of the chirps reported here should be interpreted as specific communication signals. The repetitive chirping reported here, particularly when synchronized to the S2 frequency, suggests a different function. Rather than stereotyped signals, we suggest that chirp-like frequency shifts are a component of dynamic jamming interactions. Dramatically short intervals, like those that comprise chirp signals, may be dynamically interspersed and combined with shifts in baseline frequency to sustain long periods of specific phase relationships between fish EODs.

In our limited sample of *Brachyhypopomus* chirps, we found no evidence that the chirps are specifically timed to the S2 playback. In both *Steatogenys* and *Microsternarchus*, however, dynamic changes in base frequency and inter-chirp intervals altered the phase interactions between conspecifics in structured ways that reduced jamming for the S1 and increased it for the S2. The examples shown in Figures 6–8 illustrate that specifically timed chirps can effectuate jamming (Figure 7) or jamming avoidance, although jamming was much more frequent.

This use of chirps or chirp-like behaviors as a jamming behavior has never been reported and it is not clear if we should consider these chirps reported here to be jamming avoidance behaviors, stereotyped signals or some combination of the two. To be clear, these chirps were not observed in specific interactions (i.e., courtship or male-male conflict) and the artificiality of robotic playback complicates interpretation of chirp function. It is also possible, even likely, that signal chirps occurring in a natural context are timed to the same phase relations described here. This would enhance their salience, given the low responsiveness seen during the middle of the EOD cycle. Existing descriptions of chirps in pulse species do not address this question, but we hope that future reports will examine the timing of chirps with respect to receiver phase.

### The Chirp as Startle Response in *Steatogeny*s

The short latency chirp seen frequently in *Steatogenys* is striking. Chirps often occurred with the very next interval following stimulus onset, with the very first chirp EOD occurring as soon as 18-ms following the presentation of a single S2. This short latency makes it possible that the control of these chirps is not via a thalamic prepacemaker nucleus, but rather through intrinisic hindbrain circuitry. Falconi et al. (1995) described a Mauthner cell mediated abrupt frequency increase that they interpreted as an orienting response in *G. omarum*. The so-called M-AIR (Mauthner-initiated Abrupt Increase in Rate) resembles the natural orienting response of most pulse gymnotiforms: a brief frequency increase lasting some tens of intervals. Orienting responses are the most common electromotor behaviors seen in all species of pulse gymnotiforms and are thought to provide enhanced electroreception for brief periods of attention. The overall frequency increase of orienting responses is much smaller than that of chirps, usually <25% (occurring gradually relative to chirps) over 5 or more intervals, with a longer gradual return to baseline. Curti et al. (2006) showed that Mauthner neurons initiate the M-AIR by stimulation of NMDA activation of pacemaker neurons. Based on the timing and geometry of field potentials recorded in the pacemaker nucleus, these authors inferred that the M-cell activates pacemaker cells through local medullary interneurons, possibly components of the larger M-cell circuit or a previously unrecognized medullary pre-pacemaker network described by Comas and Borde (2010). *Steatogenys* also displays a typical gymnotiform orienting response, but the short latency chirp is dramatic and is possibly related to the spontaneous chirps we have observed in isolated exploration (Field, 2016). We suggest that the Mauthner neurons of *Steatogenys*, like those of *G. omarum*, are capable of short-latency influence over the pacemaker nucleus, possibly through medullary interneurons. This may be a convergent evolution in these distantly related genera, although the medullary pre-pacemaker inputs to the pacemaker nucleus reported by Comas and Borde (2010) suggest that there may be greater complexity of inputs to the pacemaker in pulse fishes or in species that have not yet been studied. Inputs to the M-cell system are typically reflective of the sensory complement of each species (Canfield and Rose, 1996), so it should be expected that electrosensory information is part of the M-cell system inputs. Canfield and Rose (1993) showed that electrosensory inputs can modulate the directionality of typical M-cell mediated behavior (acoustically stimulated escape), so it is likely that M-cell networks in all gymnotiforms receive electroreceptive input of some form. The possibility that the M-cell network can influence pacemaker activity deserves further exploration.

### Circuit Considerations

**FIGURE 10:**
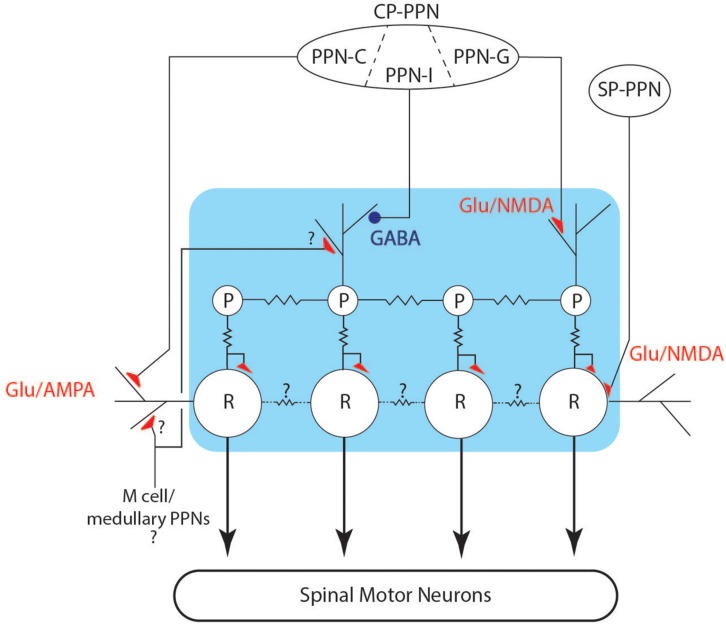
Schematic of the pacemaker control circuit. The connections between pre-pacemaker nuclei and cells of the pacemaker (blue box) are shown, including inhibitory (blue circles) and excitatory (red triangles) connections. Electrotonic coupling is indicated by the jagged lines between cells.

There are two main features of chirps, a dramatic, but short-lived increase in rate, and a deformation or reduction of the EOD waveform. Both of these effects can be understood in relation to the EOD control circuit. The neuronal network (Figure 10) controlling the modulations of the EOD rate in Gymnotiformes is well characterized generally (Dye and Meyer, 1986; Metzner, 1999; Caputi et al., 2005) and consists of an unpaired medullary pacemaker nucleus (PN) containing two cell types: pacemaker (P) and relay cells (R). Apteronotid species also have a third PN cell type, but this has not been found in other gymnotiforms (Quintana et al., 2011). P-cells are smaller (50–100 μm) and have an intrinsically rhythmic activity (Bennett, 1968). These cells are electrotonically coupled by gap junctions and are the driver of electric organ rhythm (Bennett et al., 1967). The P cells have their cell bodies and axons restrained within the PN, and their dendrites mostly occupy the dorsal portion of nucleus (Kennedy and Heiligenberg, 1994), where they are contacted by axons from pre-pacemaker nuclei in the thalamus (CP-PPN). P cell axons project to R cells, where they make mixed chemical and electrical synapses. The R cells are larger (200–300 μm) than P cells and their cell bodies are also restricted to the nucleus. Their dendrites reach the surrounding regions of the PN, where they are also contacted by pre-pacemaker axons from both CP-PPN and a sub-lemniscal prepacemaker (SP-PPN). The R cell axons descend into the spinal cord and contact local motor neurons that drive electrocyte groups within the electric organ. Under ordinary circumstances, the P and R cells are electrotonically coupled and each P action potential drives a single action potential in R cells, in turn eliciting a single discharge from a unit of spinal motor neurons and their associated electrocytes. It is well known that P cells are electronically coupled via gap junctions, but relay cells are often also inter-connected with gap junctions, albeit with large and mostly unexplored species differences (Bennett et al., 1967). A more recent anatomical study (Quintana et al., 2011) of *B. gauderio* also suggested that there may be multiple populations of relay cells, allowing for network specializations that increase the diversity of outputs (rate changes). Relay cell differentiation might also be important for the coordination of electrocyte sub-populations in patterned activation patterns (e.g., Caputi, 1999). Future comparative studies of PN organization are needed to explore these questions.

Two pre-pacemaker nuclei directly contact the PN and their synaptic actions are well studied (Kawasaki and Heiligenberg, 1988, 1989, 1990; Kennedy and Heiligenberg, 1994; Spiro, 1997). The thalamic prepacemaker nucleus (CP-PPN) is a complex of cell groups within the central posterior thalamus that sends axons to the PN where they synapse on P cell dendrites. The PPN in pulse-species is subdivided into three regions, the PPN-G, -I, and -C. Stimulation of PPN-G activates NMDA receptors on P cells and causes a smooth increase in EO frequency, with coupled R cell activity. Similarly, stimulation of PPN-I causes a smooth decrease in EO frequency (Kawasaki and Heiligenberg, 1989), in this case by activation of GABA receptors on P cells. Gymnotiform species have different dynamics to these smooth frequency changes, with frequency increases generally occurring more abruptly than decreases. Higher levels of stimulation of PPN-I lead to complete inhibition of P cell firing, resulting in silencing of relay cells and an interruption of EOD rhythm (Kawasaki and Heiligenberg, 1989, 1990; Spiro, 1997). These interruptions are different from those we recorded here, in that PPN-I stimulated interruptions begin with a smooth decrease in rate and have little or noise during the interruption. When the EOD resumes following PPN-I stimulation, EODs are fully formed as normal, and the rate gradually increases back to baseline. PPN-I stimulation does not appear to cause disruption in the coordination of R cell action potentials.

Stimulation of PPN-C causes AMPA activation on R cell receptors, and causes sustained depolarization and co-ordinated ringing in relay cells, resulting in abrupt EOD frequency increases resembling chirps (Kawasaki and Heiligenberg, 1989). With stronger stimulation of PPN-C, relay cell synchronization deteriorates; leading to amplitude and waveform modulation of the EODs within the chirp as electrocyte firing coordination degrades (Kawasaki and Heiligenberg, 1989; Spiro, 1997). Spiro (1997) reported on a limited set of simultaneous recordings from two R cells during AMPA activation and showed that some amount of R cell synchrony is maintained during early stages of the R cell depolarization. This could explain the gradual decrease of EOD amplitude through the time course of a chirp, as relay cell coordination fails with prolonged AMPA activation. The structure of chirps, tumultuous rises and interruptions all suggest that relay cell depolarization and/or ringing may contribute to the control of these behaviors.

The second major input to PN is the sublemniscal pre-pacemaker nucleus (SP-PPN) located in the midbrain. SP-PPN cells extend axons to the dendritic fields of R cells, and NMDA receptor activation of R cells causes prolonged depolarization in relay cell populations, leading to an abrupt cessation of EODs. During the depolarization, R cells fire bursts of low amplitude action potentials, leading to unsynchronized firing of the electrocytes and resulting in high frequency hash or “hushing silence,” from the electric organ (Figure 9; Spiro, 1997). P cells continue firing relatively unchanged, although back-propagated action potentials from R cells can insert extraneous R cell action potentials (Spiro, 1997). When R cells repolarize, normal firing patterns resume and the EOD rate returns at very close to the rate prior to the interruption. This pattern matches the interruptions we observed in *Microsternarchus*.

Comas and Borde (2010) also described medullary neurons retrogradely labeled following biocytin injection to the pacemaker nucleus. They described these neurons as part of medullary pre-pacemaker network, but this possibility has not been pursued in the literature. The short latency of some *Steatogenys* chirps is consistent with more local control and the high-speed dynamic nature of jamming interactions (personal observations) also suggests that local networks may play a larger role in pulse fish pacemaker modulations.

With respect to the behaviors reported here, chirps are almost certainly a result of PPN-C activation of AMPA receptors on relay cells, leading to coordinated high frequency EODs. Amplitude reduction of EODs may occur from a breakdown in synchrony between R cells. An important topic for future study is the question of R cell subpopulations and possible intra-PN mechanisms of regulating EOD amplitude during chirps (see Quintana et al., 2011). These mechanisms may lead to motivational coding in chirp expression, i.e., greater AMPA activation is specifically correlated to R cell desynchronization. Alternatively, there is also the possibility that selective recruitment of R cell sub populations could lead to patterned changes in EOD amplitude. There is no experimental evidence for the mechanism leading to specifically timed chirps or chirp like intervals, but it presumably relies on precise time-coded inputs to the PPN-C or direct connection to the pacemaker nucleus.

The short latency chirps of *Steatogenys* and the M-AIR of *Gymnotus* indicate that other inputs to the PN could exist, presumably within the hindbrain. In the case of *Gymnotus* M-AIR, these hindbrain prepacemakers exert their influence over P cells (Curti et al., 2006; Comas and Borde, 2010), but the short latency chirps in *Steatogenys* are indistinguishable from other chirps and presumably result from R cell activation. Integration of the PN with other hindbrain circuits, including the escape circuit, should be the subject of future investigation.

Interruptions reported here resemble those seen in other species and are likely to result from SPPN activation of AMPA receptors on relay cells. The tumultuous rise is the most unusual behavior and has not been reported as an outcome in physiological recordings. We speculate that it results from a combined PPN-C and PPN-G activation of both R and P cells. The frequency increase (generated by PPN-G activation of R cells) is larger than typical PPN-G mediated rate increases, but the smooth increase and sustained and steady high rate suggests changes to P cell rhythm.

### The Functions of Chirps

In this report, we have shown that chirps in *Steatogenys* and *Microsternarchus* have a wide range of EOD rate and waveform modulations. This variation in structure could reflect multiple specific types (each with some range of motivational and expressional variation), as has been shown from many Apteronotid species and more recently for *G. omarum* (Batista et al., 2012) and *B. gauderio* (Perrone et al., 2009). This variation could also reflect an absence of specificity, with chirp-like intervals dynamically used in JAR interactions or as part of a larger communication and influence system that is not based solely on specifically evolved signals (Rendall et al., 2009). The function of a communication signal is best understood from the regularity of response elicited from conspecifics and the context of the interaction between communicators. For instance, if chirps are most often displayed by the larger of a pair and the production of chirps predicts increased aggressive behavior, as in *G. carapo* (Westby, 1975) or *B. gauderio* (Perrone et al., 2009), the chirp is clearly an attack warning by the dominant partner. Or, if chirps are often displayed by the loser of a contest following retreat, as in *G. omarum* (Batista et al., 2012), then the chirp can be interpreted as a signal of submission. Perrone et al. (2009) detail the specific context of multiple chirp types in *B. gauderio* using controlled context and field observations, and more studies of this sort will be greatly informative. Their results indicate that not only can chirps vary in function between closely related species, but also multiple structural types might be deployed with distinct meanings in a single species. In addition to specifically evolved behavioral contexts, the structure of communication signals creates constraints and affordances for their functional deployment. With respect to the current findings, several possibilities exist. We list these here as possibilities, to be explored with future experimental data. Chirps in *Steatogenys* may be a component of orienting behavior, mediated by hindbrain startle and escape circuits. The chirp itself in this context could have either sensory consequences (improved sampling) or even function as an alarm signal to conspecifics. Chirps in *Steatogenys* may contain an unambiguous signal of reproductive or androgenic state and could be used in agonistic and reproductive contexts as honest indicators of state (e.g., larger chirps signal higher testosterone levels). Chirps and chirp-like intervals are used in both *Microsternarchus* and *Steatogenys* during jamming interactions and can manipulate phase relations between partners for extended periods. It is not yet clear whether this manipulation itself has signal value (i.e., is jamming an agonistic behavior?), or if the chirps involved are themselves signals as well. We look forward to future studies of chirp significance, control, and diversity across pulse gymnotiforms. The jamming avoidance interactions described here suggest that jamming interactions comprise a dynamic communication system that is both a mechanism of preserving private electrolocation abilities and also system of intraspecific influence and communication.

## Data Availability Statement

The datasets generated for this study are available on request to the corresponding author.

## Ethics Statement

The animal study was reviewed and approved by Hunter College Institutional Animal Care and Use Committee and the Ethical Committee for Animal Research of INPA.

## Author Contributions

All authors contributed to the design of the experiments, led by CB. CF and TP conducted the experiments and collected the data. CB, JA-G, and TP developed the ethographic framework for the description of specific electromotor behaviors like the chirp and tumultuous rise. CB and TP designed and conducted the statistical analysis. CB prepared the final version of the manuscript and illustrations, with contributions and comments from all authors.

## Conflict of Interest

The authors declare that the research was conducted in the absence of any commercial or financial relationships that could be construed as a potential conflict of interest.

## Supplementary Material

FIGURE S1 Beeswarm plots of the three most prolific chirpers in *Steatogenys* in dF experiments **(A)** and S2 duration experiments **(B)**. The three most prolific chirpers in *Microsternarchus* are shown in **(C)**. As in Figure 2, the top plot of each panel shows the reduction of IPI as a function of position within the chirp and the bottom plot shows the reduction of amplitude for each successive EOD within the chirp.

FIGURE S2 Distributions of trial mean chirp starts grouped by the individuals with significant overall distributions. **(A)** Six individual *Microsternarchus* had significantly concentrated distributions of chirp start angles. **(B)** Fifteen individual *Steatogenys* had significantly directed distributions. In all panels, the number of chirps, mean vector, and *r*-score are shown above the histograms. *P* < 0.05 for all instances shown.

TABLE S1 Summary statistics of chirp metrics for each subject. **(a)** *Steatogenys*. **(b)** *Microsternarchus*. **(c)** *Brachyhypopomus*.
