# Supplementary figures and images for: A JAR of Chirps: The Gymnotiform Chirp Can Function as Both a Communication Signal and a Jamming Avoidance Response
Source: Front Integr Neurosci. 2019 Oct 2;13:55. doi: 10.3389/fnint.2019.00055 (PMC6783576; doi:10.3389/fnint.2019.00055)

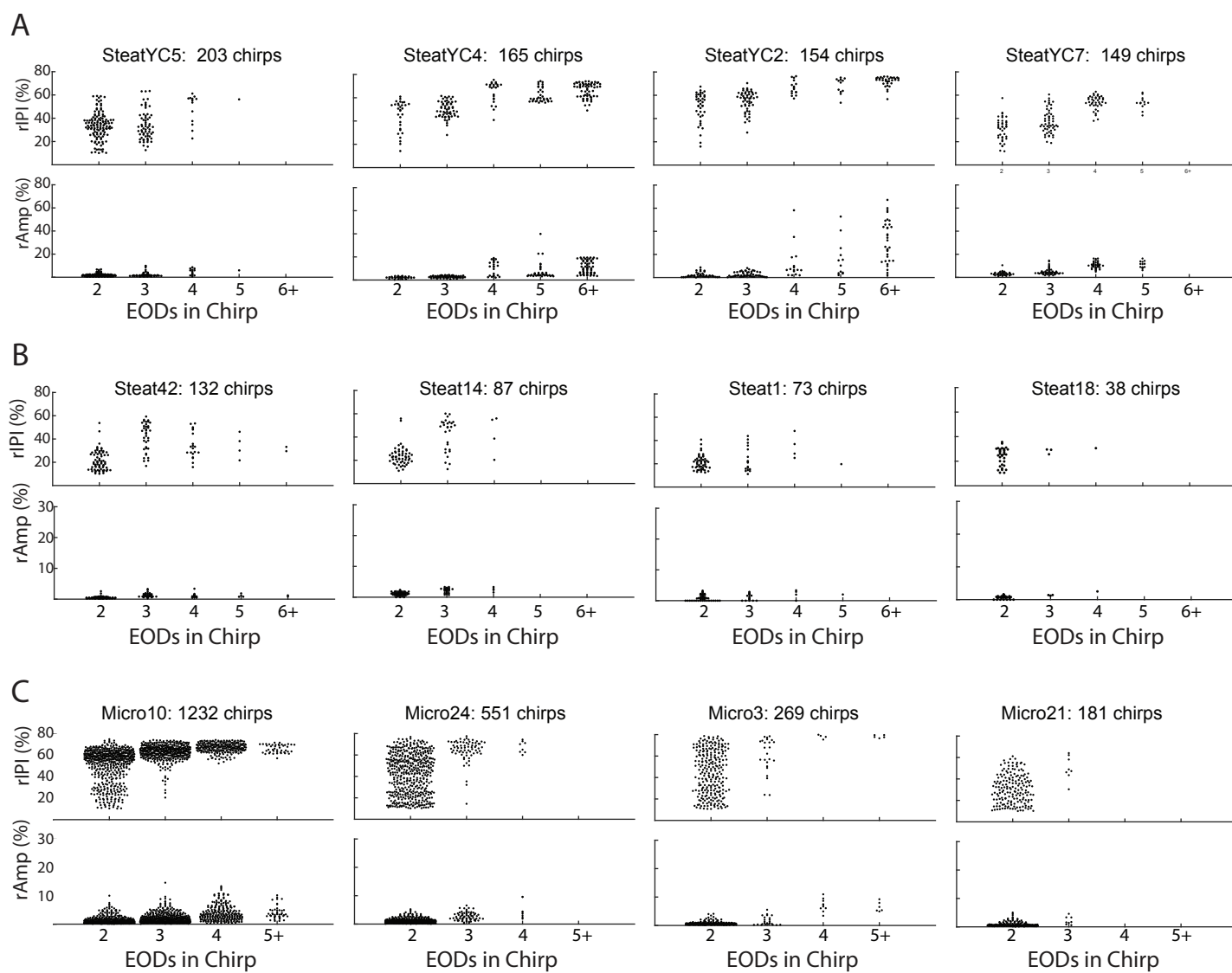

Supplement: FIGURE S1 — Beeswarm plots of the three most prolific chirpers in Steatogenys in dF experiments (A) and S2 duration experiments (B). The three most prolific chirpers in Microsternarchus are shown in (C). As in Figure 2, the top plot of each panel shows the reduction of IPI as a function of position within the chirp and the bottom plot shows the reduction of amplitude for each successive EOD within the chirp. [file Image_1.pdf]

A

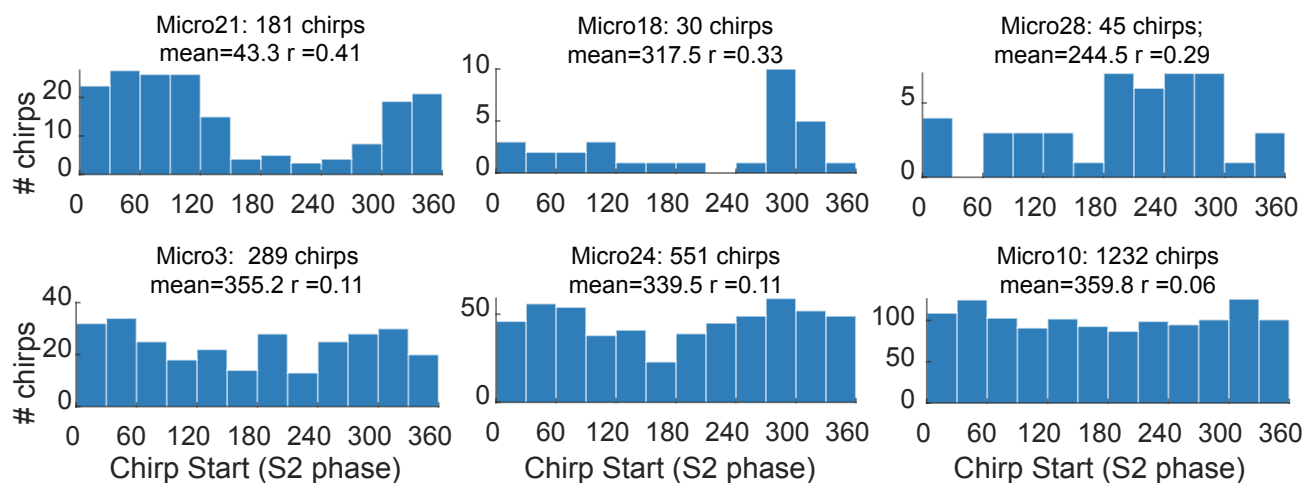

B

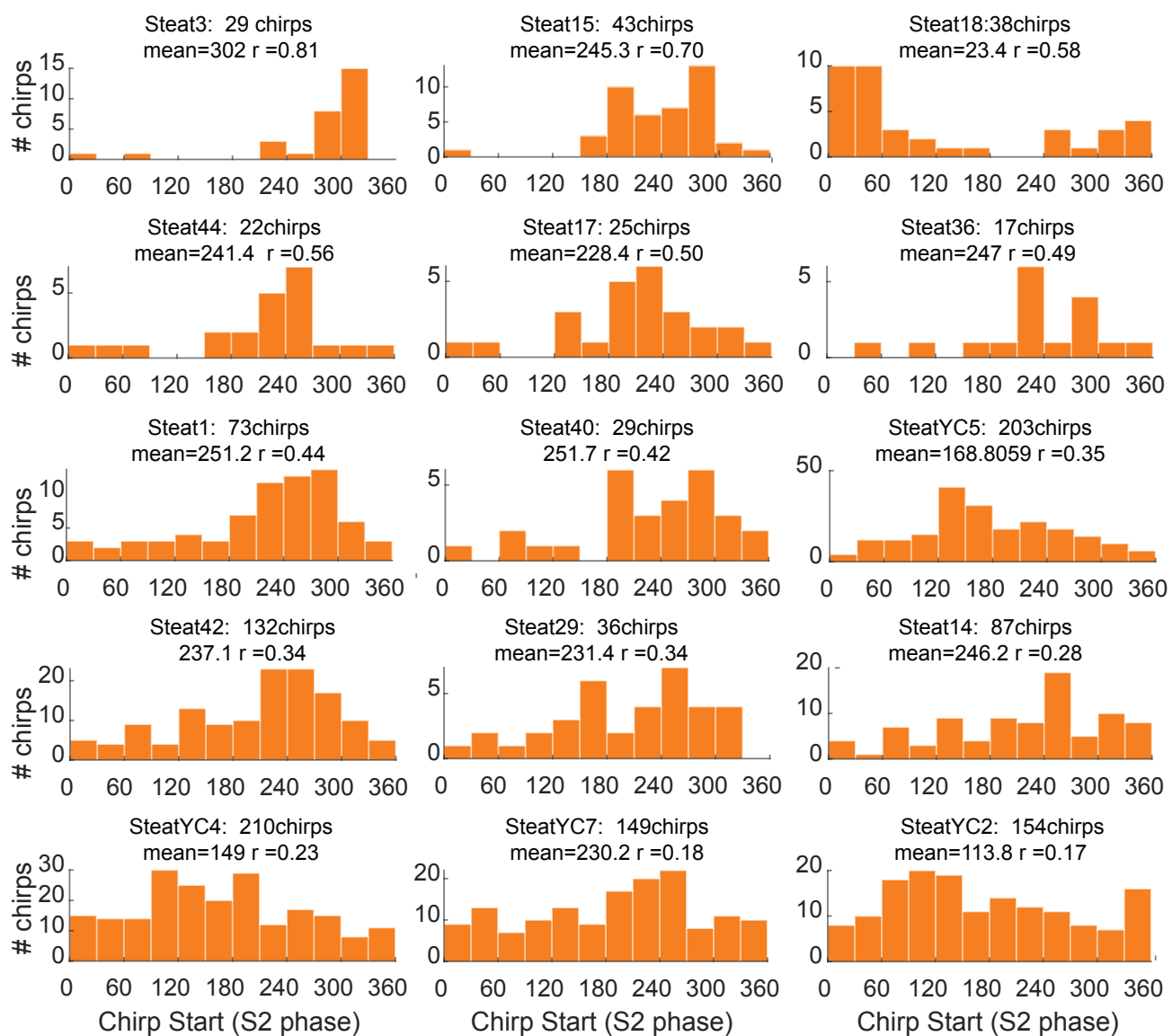

Supplement: FIGURE S2 — Distributions of trial mean chirp starts grouped by the individuals with significant overall distributions. (A) Six individual Microsternarchus had significantly concentrated distributions of chirp start angles. (B) Fifteen individual Steatogenys had significantly directed distributions. In all panels, the number of chirps, mean vector, and r-score are shown above the histograms. P < 0.05 for all instances shown. [file Image_2.pdf]
