# Supplementary material for: A JAR of Chirps: The Gymnotiform Chirp Can Function as Both a Communication Signal and a Jamming Avoidance Response
Source: Front Integr Neurosci. 2019 Oct 2;13:55. doi: 10.3389/fnint.2019.00055 (PMC6783576; doi:10.3389/fnint.2019.00055)
Supplement: TABLE S1 — Summary statistics of chirp metrics for each subject. (a) Steatogenys. (b) Microsternarchus. (c) Brachyhypopomus. [file Data_Sheet_1.pdf]

a.

			Chirp Metrics -- **Mean** (SD)<br>*(min - max)*						
			Duration						
	Subject ID	# chirps	EODs in chirp	time (s)	# S2 cycles	starting phase (°)	rIPI (%)	pkF (Hz)	rAmp (%)
Steatogenys	Steat001	73	**2.384** (0.659)<br>*(2 - 5)*	**0.040** (0.014)<br>*(0.025 - 0.119)*	**2.128** (0.685)<br>*(1.508 - 5.517)*	**251.176** (84.162)<br>*(9.345 - 358.632)*	**22.390** (8.106)<br>*(11.158 - 48.192)*	**71.186** (9.246)<br>*(58.407 - 101.725)*	**1.103** (1.021)<br>*(0.000 - 3.350)*
	Steat011	6	**2.333** (0.516)<br>*(2 - 3)*	**0.035** (0.013)<br>*(0.026 - 0.059)*	**2.270** (0.928)<br>*(1.575 - 3.926)*	**302.845** (124.136)<br>*(1.477 - 327.185)*	**15.706** (4.815)<br>*(11.569 - 24.644)*	**77.848** (5.658)<br>*(73.426 - 87.979)*	**0.993** (0.685)<br>*(0.000 - 1.834)*
	Steat012	2	**2** (0)<br>*(2 - 2)*	**0.031** (0.004)<br>*(0.029 - 0.034)*	**1.776** (0.101)<br>*(1.705 - 1.847)*	**320.040** (38.485)<br>*(292.827 - 347.253)*	**26.303** (21.448)<br>*(11.137 - 41.469)*	**81.293** (24.818)<br>*(63.744 - 98.842)*	**1.019** (0.892)<br>*(0.388 - 1.649)*
	Steat014	87	**2.414** (0.582)<br>*(2 - 4)*	**0.040** (0.008)<br>*(0.027 - 0.071)*	**1.974** (0.418)<br>*(1.288 - 3.562)*	**246.152** (91.345)<br>*(3.651 - 352.296)*	**30.465** (13.990)<br>*(10.974 - 60.142)*	**74.959** (18.742)<br>*(55.996 - 125.522)*	**1.527** (0.812)<br>*(0.000 - 3.305)*
	Steat015	43	**2.302** (0.465)<br>*(2 - 3)*	**0.036** (0.009)<br>*(0.027 - 0.063)*	**1.991** (0.510)<br>*(1.412 - 3.822)*	**245.299** (57.312)<br>*(4.030 - 337.887)*	**27.418** (10.061)<br>*(10.164 - 44.408)*	**78.560** (11.241)<br>*(59.402 - 102.580)*	**0.453** (0.291)<br>*(0.000 - 1.706)*
	Steat017	25	**2.52** (0.770)<br>*(2 - 4)*	**0.041** (0.019)<br>*(0.025 - 0.105)*	**2.285** (1.101)<br>*(1.463 - 6.101)*	**228.409** (78.067)<br>*(11.143 - 356.727)*	**26.874** (11.616)<br>*(10.693 - 52.069)*	**81.253** (14.120)<br>*(58.758 - 109.480)*	**1.622** (0.682)<br>*(0.595 - 3.197)*
	Steat018	38	**2.132** (0.414)<br>*(2 - 4)*	**0.041** (0.019)<br>*(0.031 - 0.112)*	**2.045** (0.912)<br>*(1.380 - 5.379)*	**23.440** (123.969)<br>*(3.296 - 353.994)*	**24.406** (6.938)<br>*(10.561 - 35.633)*	**68.858** (4.508)<br>*(55.173 - 78.125)*	**0.818** (0.550)<br>*(0.000 - 2.495)*
	Steat024	10	**2.2** (0.422)<br>*(2 - 3)*	**0.033** (0.009)<br>*(0.026 - 0.055)*	**2.054** (0.542)<br>*(1.597 - 3.365)*	**269.901** (33.583)<br>*(195.672 - 304.330)*	**21.311** (9.477)<br>*(12.276 - 40.759)*	**80.657** (11.214)<br>*(71.178 - 104.334)*	**0.997** (0.341)<br>*(0.647 - 1.811)*
	Steat029	36	**2.444** (0.695)<br>*(2 - 4)*	**0.037** (0.012)<br>*(0.024 - 0.073)*	**2.156** (0.708)<br>*(1.453 - 4.450)*	**231.403** (84.685)<br>*(6.767 - 326.977)*	**27.723** (11.525)<br>*(11.578 - 53.146)*	**85.270** (19.036)<br>*(61.652 - 139.509)*	**0.936** (0.422)<br>*(0.282 - 2.385)*
	Steat003	29	**2** (0)<br>*(2 - 2)*	**0.029** (0.001)<br>*(0.027 - 0.030)*	**1.834** (0.096)<br>*(1.605 - 2.027)*	**302.008** (70.590)<br>*(22.618 - 327.360)*	**13.266** (3.739)<br>*(10.286 - 26.755)*	**74.152** (3.816)<br>*(70.765 - 88.297)*	**1.002** (0.970)<br>*(0.000 - 3.018)*
	Steat036	17	**2.059** (0.243)<br>*(2 - 3)*	**0.030** (0.003)<br>*(0.027 - 0.040)*	**1.842** (0.187)<br>*(1.623 - 2.426)*	**247.027** (74.916)<br>*(53.858 - 336.063)*	**15.571** (3.733)<br>*(10.506 - 22.585)*	**75.093** (4.925)<br>*(69.064 - 84.042)*	**0.532** (0.237)<br>*(0.290 - 1.183)*
	Steat040	29	**2.241** (0.435)<br>*(2 - 3)*	**0.036** (0.006)<br>*(0.028 - 0.048)*	**1.937** (0.322)<br>*(1.288 - 2.519)*	**251.744** (83.983)<br>*(18.038 - 352.250)*	**25.577** (12.112)<br>*(11.914 - 51.742)*	**73.258** (13.350)<br>*(60.506 - 106.845)*	**1.755** (0.632)<br>*(0.487 - 2.957)*
	Steat042	132	**2.742** (0.962)<br>*(2 - 7)*	**0.046** (0.018)<br>*(0.025 - 0.135)*	**2.342** (0.928)<br>*(1.303 - 6.529)*	**237.099** (84.500)<br>*(10.701 - 348.246)*	**29.662** (13.548)<br>*(10.127 - 59.294)*	**76.652** (17.324)<br>*(56.909 - 124.562)*	**0.826** (0.614)<br>*(0.133 - 3.386)*
	Steat044	22	**2.409** (0.590)<br>*(2 - 4)*	**0.043** (0.012)<br>*(0.032 - 0.071)*	**2.051** (0.590)<br>*(1.324 - 3.512)*	**241.428** (79.885)<br>*(19.390 - 337.592)*	**27.110** (9.400)<br>*(12.463 - 41.108)*	**68.794** (9.277)<br>*(55.173 - 87.506)*	**0.323** (0.226)<br>*(0.000 - 0.698)*
	SteatYC1	82	**4.537** (1.557)<br>*(2 - 8)*	**0.057** (0.021)<br>*(0.028 - 0.144)*	**2.710** (0.987)<br>*(1.361 - 7.070)*	**22.254** (112.074)<br>*(4.202 - 355.636)*	**69.737** (4.748)<br>*(51.478 - 79.872)*	**159.787** (27.501)<br>*(102.580 - 268.286)*	**23.053** (16.190)<br>*(4.197 - 87.989)*
	SteatYC2	154	**3.916** (2.048)<br>*(2 - 11)*	**0.046** (0.018)<br>*(0.023 - 0.121)*	**2.597** (1.235)<br>*(1.293 - 8.376)*	**113.791** (98.916)<br>*(9.317 - 359.172)*	**59.571** (12.418)<br>*(15.892 - 76.371)*	**149.666** (49.173)<br>*(67.535 - 254.313)*	**10.832** (15.840)<br>*(0.108 - 67.192)*
	SteatYC3	1	**2** (0)<br>*(2 - 2)*	**0.025** (0.000)<br>*(0.025 - 0.025)*	**1.629** (0.000)<br>*(1.629 - 1.629)*	**135.831** (0.000)<br>*(135.831 - 135.831)*	**42.726** (0.000)<br>*(42.726 - 47.726)*	**111.735** (0.000)<br>*(111.735 - 111.735)*	**3.152** (0.000)<br>*(3.152 - 3.152)*
	SteatYC4	165	**4.103** (1.625)<br>*(2 - 9)*	**0.049** (0.021)<br>*(0.023 - 0.134)*	**2.906** (1.241)<br>*(1.378 - 8.717)*	**127.721** (93.704)<br>*(3.880 - 357.046)*	**56.411** (11.603)<br>*(14.216 - 75.377)*	**145.312** (38.148)<br>*(69.754 - 242.926)*	**6.104** (5.575)<br>*(0.660 - 39.899)*
	SteatYC5	203	**2.453** (0.638)<br>*(2 - 5)*	**0.033** (0.010)<br>*(0.022 - 0.087)*	**2.068** (0.605)<br>*(1.421 - 5.091)*	**168.806** (79.657)<br>*(15.193 - 356.119)*	**34.718** (12.442)<br>*(10.120 - 63.368)*	**100.807** (21.914)<br>*(69.655 - 171.327)*	**2.172** (1.754)<br>*(0.358 - 9.850)*
	SteatYC7	149	**3.168** (0.933)<br>*(2 - 5)*	**0.042** (0.011)<br>*(0.024 - 0.069)*	**2.464** (0.608)<br>*(1.387 - 3.988)*	**230.222** (96.269)<br>*(1.708 - 359.159)*	**41.341** (12.896)<br>*(11.779 - 62.954)*	**102.707** (22.168)<br>*(67.442 - 152.113)*	**6.486** (4.083)<br>*(0.956 - 16.362)*
	SteatYC8	11	**2.455** (0.688)<br>*(2 - 4)*	**0.033** (0.008)<br>*(0.024 - 0.054)*	**2.265** (0.528)<br>*(1.871 - 3.529)*	**316.696** (92.523)<br>*(65.650 - 339.332)*	**21.553** (10.354)<br>*(10.139 - 40.398)*	**86.500** (15.857)<br>*(72.878 - 116.535)*	**0.682** (0.545)<br>*(0.104 - 1.904)*
		**M = 62.571**<br>**SD = 62.092**	**M = 2.610**<br>**SD = 0.718**	**M = 0.038**<br>**SD = 0.007**	**M = 2.158**<br>**SD = 0.317**	**M = 214.918**<br>**SD = 85.798**	**M = 31.421**<br>**SD = 14.855**	**M = 91.636**<br>**SD = 27.519**	**M = 3.161**<br>**SD = 5.241**

Field et al Table S2A (supplemental)
