# Supplementary material for: A JAR of Chirps: The Gymnotiform Chirp Can Function as Both a Communication Signal and a Jamming Avoidance Response
Source: Front Integr Neurosci. 2019 Oct 2;13:55. doi: 10.3389/fnint.2019.00055 (PMC6783576; doi:10.3389/fnint.2019.00055)
Supplement: Supplementary file 4 [file Data_Sheet_2.pdf]

b.

	Subject ID	# chirps	Duration: EODs in chirp	Duration: time (s)	Duration: # S2 cycles	starting phase (°)	rIPI (%)	pkF (Hz)	rAmp (%)
Microsternarchus	Micro010	1232	**2.825** (0.865) *(2 - 6)*	**0.023** (0.006) *(0.014 - 0.105)*	**1.983** (0.548) *(1.090 - 7.992)*	359.853 (107.204) (0.000 - 359.344)	**58.810** (13.189) *(10.407 - 75.340)*	**224.623** (52.755) *(89.758 - 341.455)*	**2.145** (2.157) *(0.063 - 14.657)*
	Micro011	5	**2.600** (0.894) *(2 - 4)*	**0.031** (0.002) *(0.028 - 0.033)*	**1.762** (0.128) *(1.640 - 1.973)*	297.810 (81.683) (115.447 - 307.873)	**42.697** (35.660) *(12.629 - 82.574)*	**161.239** (129.387) *(64.163 - 319.138)*	**5.609** (7.577) *(0.279 - 17.313)*
	Micro012	13	**2.077** (0.277) *(2 - 3)*	**0.031** (0.007) *(0.019 - 0.040)*	**1.564** (0.396) *(0.815 - 2.050)*	129.223 (99.573) (2.785 - 341.728)	**46.404** (23.371) *(11.569 - 76.983)*	**117.448** (60.992) *(54.133 - 213.223)*	**0.693** (0.601) *(0.187 - 2.074)*
	Micro018	30	**2.067** (0.254) *(2 - 3)*	**0.041** (0.017) *(0.030 - 0.123)*	**1.927** (0.705) *(1.177 - 5.444)*	317.459 (112.079) (2.704 - 337.206)	**29.698** (14.704) *(14.848 - 64.253)*	**67.749** (19.345) *(47.544 - 126.826)*	**0.499** (0.238) *(0.110 - 0.981)*
	Micro019	33	**2.121** (0.331) *(2 - 3)*	**0.028** (0.004) *(0.022 - 0.037)*	**1.891** (0.234) *(1.536 - 2.529)*	48.377 (106.734) (9.191 - 331.404)	**39.931** (18.819) *(11.306 - 72.186)*	**114.672** (43.540) *(60.431 - 227.108)*	**0.724** (0.717) *(0.116 - 3.992)*
	Micro021	181	**2.044** (0.206) *(2 - 3)*	**0.026** (0.003) *(0.020 - 0.038)*	**1.913** (0.192) *(1.443 - 2.809)*	43.336 (116.753) (1.649 - 353.465)	**31.696** (13.791) *(10.061 - 64.106)*	**107.318** (25.335) *(72.769 - 189.993)*	**0.953** (0.898) *(0.055 - 5.035)*
	Micro024	551	**2.154** (0.400) *(2 - 4)*	**0.031** (0.007) *(0.019 - 0.126)*	**1.801** (0.426) *(1.119 - 6.792)*	339.527 (109.374) (0.000 - 358.508)	**45.537** (19.291) *(10.204 - 77.584)*	**116.041** (42.633) *(53.894 - 244.141)*	**1.302** (1.188) *(0.059 - 9.603)*
	Micro025	87	**2.138** (0.408) *(2 - 4)*	**0.034** (0.008) *(0.021 - 0.083)*	**1.473** (0.393) *(0.852 - 3.378)*	307.854 (109.398) (11.106 - 356.828)	**45.847** (21.546) *(10.023 - 79.156)*	**106.864** (46.934) *(49.222 - 242.926)*	**1.048** (1.565) *(0.050 - 9.887)*
	Micro028	45	**2.111** (0.318) *(2 - 3)*	**0.029** (0.005) *(0.021 - 0.042)*	**1.438** (0.248) *(1.002 - 2.065)*	244.468 (94.086) (8.679 - 355.684)	**45.695** (18.852) *(12.627 - 76.123)*	**118.501** (43.622) *(63.004 - 223.982)*	**1.897** (1.527) *(0.140 - 6.403)*
	Micro003	289	**2.215** (0.603) *(2 - 5)*	**0.033** (0.011) *(0.022 - 0.140)*	**1.767** (0.580) *(1.180 - 8.243)*	355.157 (110.960) (0.000 - 358.977)	**47.996** (21.231) *(10.714 - 82.704)*	**125.509** (57.281) *(54.073 - 285.545)*	**1.105** (1.650) *(0.058 - 10.964)*
	Micro007	5	**2.200** (0.447) *(2 - 3)*	**0.033** (0.009) *(0.024 - 0.047)*	**1.813** (0.454) *(1.342 - 2.568)*	301.826 (89.244) (102.449 - 330.729)	**23.499** (17.271) *(11.758 - 53.699)*	**82.195** (29.677) *(60.807 - 134.513)*	**0.815** (0.800) *(0.215 - 2.031)*
	Micro009	84	**2.226** (0.475) *(2 - 4)*	**0.032** (0.006) *(0.023 - 0.054)*	**1.642** (0.303) *(1.177 - 2.698)*	54.913 (107.377) (5.047 - 358.750)	**45.728** (16.587) *(10.390 - 74.946)*	**112.186** (39.178) *(58.971 - 210.466)*	**1.244** (1.667) *(0.132 - 7.468)*
		**M = 212.917 SD = 358.350**	**M = 2.231 SD = 0.237**	**M = 0.031 SD = 0.005**	**M = 1.748 SD = 0.181**	M = 233.317 SD = 126.746	**M = 41.961 SD = 9.522**	**M = 121.195 SD = 39.619**	**M = 1.503 SD = 1.380**

c.

	Subject ID	# chirps	Duration: EODs in chirp	Duration: time (s)	Duration: # S2 cycles	starting phase (°)	rIPI (%)	pkF (Hz)	rAmp (%)
Brachyhypopomus	Brachy006	18	**19.667** (9.159) *(6 - 38)*	**0.170** (0.043) *(0.083 - 0.256)*	**5.3878** (2.051) *(2.342 - 9.627)*	105.332 (92.952) (60.100 - 356.085)	**79.813** (9.140) *(52.261 - 88.871)*	**187.872** (44.175) *(128.495 - 256.990)*	**15.678** (14.096) *(0.743 - 39.414)*
	Brachy008	2	**21.000** (8.485) *(15 - 27)*	**0.217** (0.005) *(0.213 - 0.220)*	**4.308** (2.037) *(2.868 - 5.749)*	246.052 (105.880) (171.183 - 320.920)	**87.812** (3.774) *(85.143 - 90.481)*	**188.488** (62.263) *(144.462 - 232.515)*	**27.888** (36.146) *(2.329 - 53.447)*
	Brachy009	38	**22.737** (8.150) *(7 - 36)*	**0.193** (0.062) *(0.048 - 0.329)*	**4.160** (1.473) *(0.997 - 8.376)*	100.903 (101.102) (2.829 - 352.976)	**83.168** (9.716) *(50.195 - 92.937)*	**212.748** (39.178) *(58.971 - 210.466)*	**33.057** (11.018) *(20.833 - 64.109)*
		**M = 19.333 SD = 18.037**	**M = 21.135 SD = 1.540**	**M = 0.193 SD = 0.023**	**M = 4.619 SD = 0.670**	**M = 150.762 SD = 82.553**	**M = 83.598 SD = 4.017**	**M = 196.370 SD = 14.188**	**M = 25.541 SD = 8.924**

Field Et al: Table S2 B&C (supplementary)
